# A metabolic biomarker predicts Parkinson’s disease at the early stages in patients and animal models

**DOI:** 10.1172/JCI146400

**Published:** 2022-02-15

**Authors:** David Mallet, Thibault Dufourd, Mélina Decourt, Carole Carcenac, Paola Bossù, Laure Verlin, Pierre-Olivier Fernagut, Marianne Benoit-Marand, Gianfranco Spalletta, Emmanuel L. Barbier, Sebastien Carnicella, Véronique Sgambato, Florence Fauvelle, Sabrina Boulet

**Affiliations:** 1 University Grenoble Alpes, INSERM, U1216, Grenoble Institut Neurosciences, Grenoble, France.; 2 Université de Poitiers, INSERM U1084, Laboratoire de Neurosciences Expérimentales et Cliniques, Poitiers, France.; 3 Dipartimento di Neurologia Clinica e Comportamentale, Laboratorio di Neuropsicobiologia Sperimentale, IRCCS Santa Lucia Foundation, Rome, Italy.; 4 University Grenoble Alpes, INSERM, US17, CNRS, UMS 3552, CHU Grenoble Alpes, IRMaGe, Grenoble, France.; 5 Laboratory of Neuropsychiatry, IRCCS Santa Lucia Foundation, Rome, Italy.; 6 Université de Lyon, CNRS UMR5229, Institut des Sciences Cognitives Marc Jeannerod, Bron, France.

**Keywords:** Metabolism, Neuroscience, Diagnostics, Parkinson disease

## Abstract

**Background:**

Care management of Parkinson’s disease (PD) patients currently remains symptomatic, mainly because diagnosis relying on the expression of the cardinal motor symptoms is made too late. Earlier detection of PD therefore represents a key step for developing therapies able to delay or slow down its progression.

**Methods:**

We investigated metabolic markers in 3 different animal models of PD, mimicking different phases of the disease assessed by behavioral and histological evaluation, and in 3 cohorts of de novo PD patients and matched controls (*n* = 129). Serum and brain tissue samples were analyzed by nuclear magnetic resonance spectroscopy and data submitted to advanced multivariate statistics.

**Results:**

Our translational strategy reveals common metabolic dysregulations in serum of the different animal models and PD patients. Some of them were mirrored in the tissue samples, possibly reflecting pathophysiological mechanisms associated with PD development. Interestingly, some metabolic dysregulations appeared before motor symptom emergence and could represent early biomarkers of PD. Finally, we built a composite biomarker with a combination of 6 metabolites. This biomarker discriminated animals mimicking PD from controls, even from the first, nonmotor signs and, very interestingly, also discriminated PD patients from healthy subjects.

**Conclusion:**

From our translational study, which included 3 animal models and 3 de novo PD patient cohorts, we propose a promising biomarker exhibiting a high accuracy for de novo PD diagnosis that may possibly predict early PD development, before motor symptoms appear.

**Funding:**

French National Research Agency (ANR), DOPALCOMP, Institut National de la Santé et de la Recherche Médicale, Université Grenoble Alpes, Association France Parkinson.

## Graphical abstract

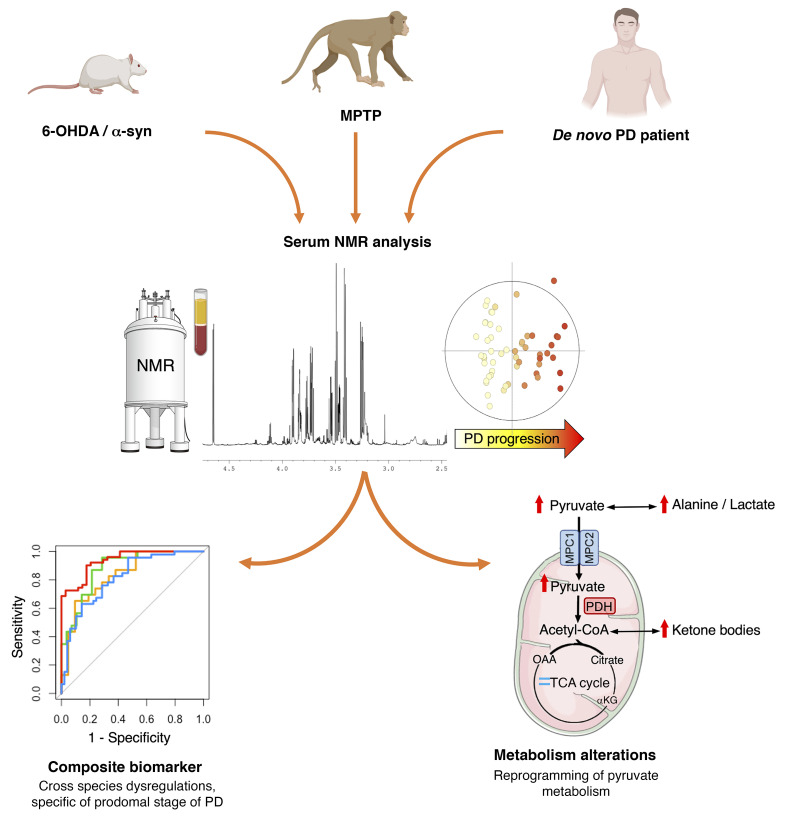

## Introduction

Parkinson’s disease (PD) is one of the most prevalent neurodegenerative diseases in the world, affecting about 1% of adults older than 60 years (1). From its onset, the pathology evolves progressively and continuously according to 3 stages, i.e., preclinical, prodromal, and clinical, corresponding, respectively, to the asymptomatic onset of neurodegeneration, nonmotor symptoms, and motor symptoms (2), finally allowing the diagnosis to be made (3). Unfortunately, the motor symptoms appear when 70% to 80% of the nigrostriatal dopamine system is already lost (1), precluding any potential neuroprotective intervention (4). Furthermore, postmortem and histopathological analyses show that the accuracy of current PD diagnosis is only 53% in patients with motor symptoms occurring for less than 5 years (5). In the early phases of PD, the prodromal phase is characterized by a panel of nonmotor symptoms, including neuropsychiatric disorders, such as apathy, which could schematically be defined as a loss of motivation (6), observed in up to 70% of PD patients (7). Although these symptoms are often premonitory of PD, they are not specific and therefore cannot be considered as predictive criteria. Thus, finding reliable, specific, and highly predictive biomarkers of the prodromal stage of the disease is a substantial challenge for the success of PD curative or preventive strategies and for the development of therapies able to delay or slow down PD progression (8, 9).

PD is a multifactorial disease (10) resulting from a complex interplay between genetic and environmental factors. The metabolome — the global pool of metabolites — reflects the interactions among genotype, lifestyle, diet, drug therapy, and environmental exposure (11). Therefore, metabolomics, i.e., metabolome analysis, could represent a powerful tool for elucidating the molecular mechanisms involved in PD and identifying potential predictive biomarkers (12–14). Metabolic alterations have already been described in patients expressing neuropsychiatric symptoms similar to those observed in the prodromal stage of PD (15). Additionally, it has been shown that the metabolic dysregulation observed in serum can accurately discriminate newly diagnosed PD patients from controls (16). Similarly, alterations in plasma metabolome have been correlated with disease progression in PD patients (17). However, the use of metabolomics as a predictive tool during the prodromal stage remains to be investigated.

**Figure 1:**
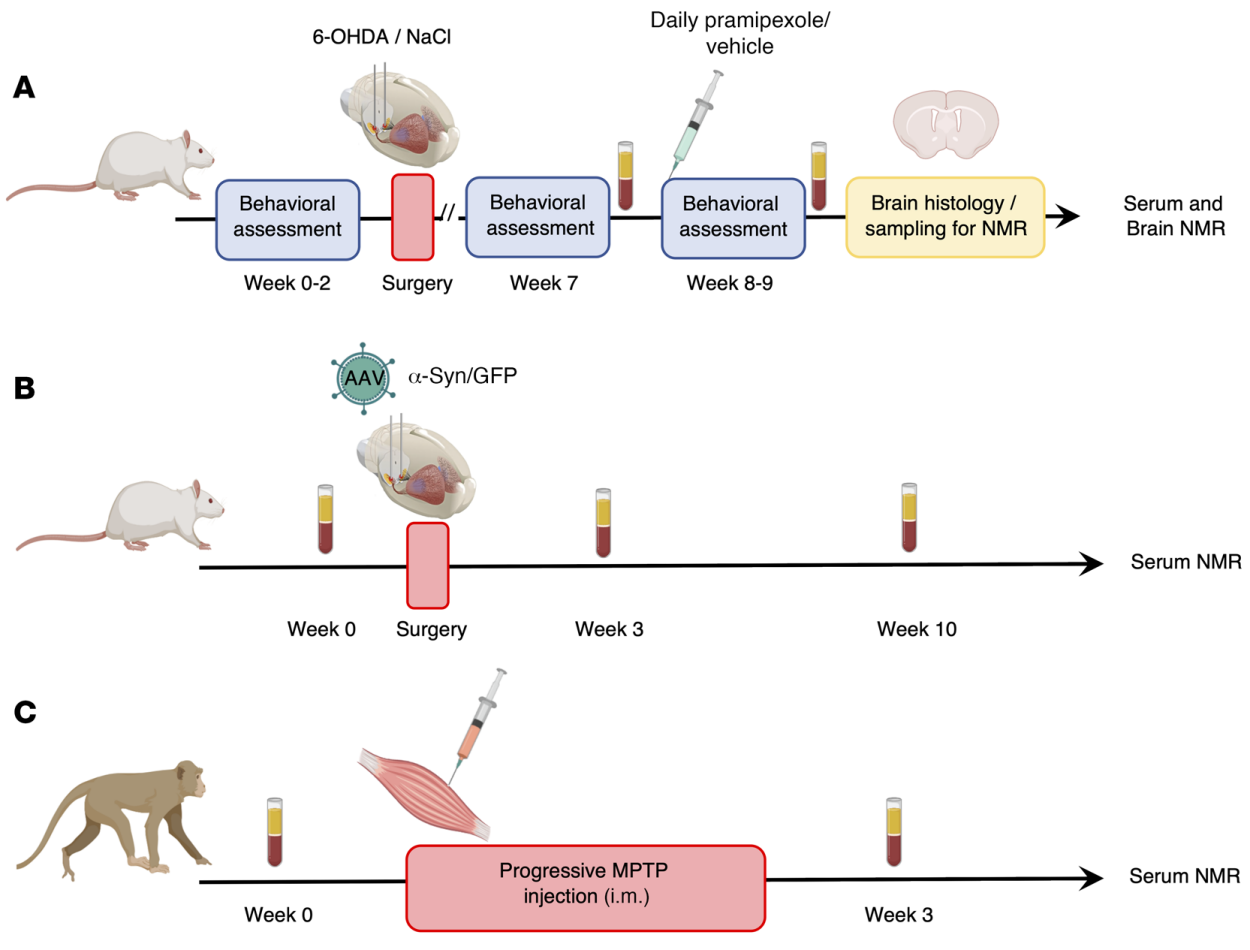
Flowchart of experimental procedures applied to different PD animal models. (**A**–**C**) All serum samples were collected after fasting and following the same protocol. They were analyzed by ^1^H-NMR at 950 MHz. Brain samples of 6-OHDA rats were used for histology and metabolomic analysis performed by ^1^H HRMAS NMR at 500 MHz.

To study this prodromal stage, animal models of PD have been widely developed (18), but none is currently able to recapitulate all phenotypic and etiological characteristics of the disease. We therefore investigated potential metabolic changes in 3 different animal models, namely, 2 complementary rodent models and 1 nonhuman primate model expressing complementary PD characteristics (19–21). We first used the 6-hydroxydopamine (6-OHDA) rat model, as it represents a gold standard and phenotypically correct model of PD, with each animal stably mimicking a specific stage of the disease, including the prodromal phase, without temporal evolution (18, 19). This allows capture of the nature of the disorder in a between-group design at a given time, similarly to a clinical study comparing PD patients at different stages of the disease. Moreover, it has good predictive value regarding the treatments classically used in clinics, such as dopaminergic agonists (22). We then used a viral vector–induced α-synuclein rat model that presents progressive synucleinopathy reflecting the progressive nature of PD in a within-group design. Nonetheless, this model does not encompass all the neuropsychiatric symptoms characteristic of the prodromal stage of PD (e.g., apathy; ref. 23). Finally, we used the nonhuman 1-methyl-4-phenyl-1,2,3,6-tetrahydropyridine (MPTP) primate model, which shares greater genome sequence identity with humans and neuroanatomical similarities (Figure 1). Metabolic changes were analyzed in blood (serum) to find biomarkers that are easily transposable to the clinic and in specific brain regions differently affected by the neurodegenerative process (i.e., the dorsal striatum [DS] and nucleus accumbens [Nacc]) to potentially associate these biomarkers with the pathophysiological processes involved in PD. We thereby built a blood biomarker from a combination of metabolites that might be relevant to the prodromal stage. From a translational perspective and in order to validate the clinical relevance of our PD biomarker candidate, we compared our preclinical results with the metabolomic results of de novo PD patients from 2 different biobanks.

## Results

### The 6-OHDA rat model allows the study of different stages of PD.

**Figure 2:**
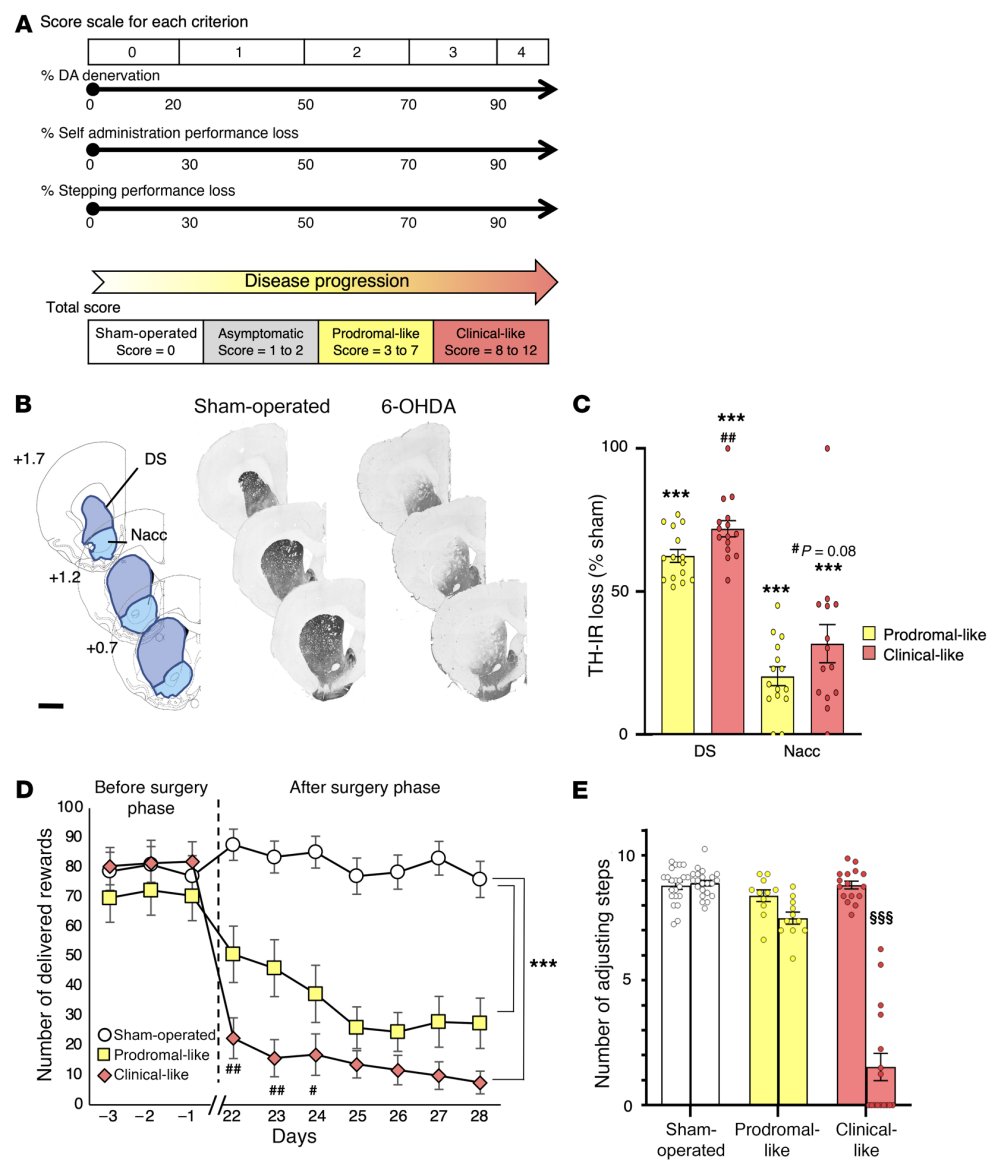
Striatal dopaminergic denervation induced by bilateral 6-OHDA lesion of the SNc leads to apathetic-like behavior and fine motor dysfunctions. (**A**) A PDP total score, as the sum of histological and behavioral components, was assigned to each animal, from 0 (sham-operated animal) to 3 to 7 (prodromal-like animal) or 8 to 12 (clinical-like animal). (**B**) Examples of TH-stained coronal sections of sham-operated and 6-OHDA rat brains at 3 striatal levels with respect to bregma and corresponding diagrams selected from the atlas of Paxinos and Watson (79), with areas used for quantification of dopaminergic denervation. Scale bar: 2 mm. (**C**) Quantification of TH-IR staining loss at the striatal levels shown in **B**, expressed as a percentage of the mean value obtained for sham-operated animals (*n* = 22). We observed a large decrease of TH-positive neurons in DS and a lighter decrease in Nacc of prodromal (*n* = 14) and clinical-like (*n* = 15) rats. (**D**) 6-OHDA SNc lesion induced an abrupt instrumental deficit in an operant sucrose self-administration procedure. Results are expressed as the mean number of sucrose deliveries per session. Sham-operated rats (*n* = 22); prodromal-like rats (*n* = 14); clinical-like rats (*n* = 15). (**E**) 6-OHDA SNc lesion reduced the number of adjusting steps in a stepping procedure only in clinical-like animals. Results are expressed as the mean number of forelimb adjustments for 2 trials before (left bars) and after (right bars) 6-OHDA (prodromal-like (*n* = 14) and clinical-like (*n* = 15) animals or saline (sham) injection (*n* = 22). Data are presented as mean ± SEM and were determined by 1-way ANOVA or RM-ANOVA followed by Tukey’s post hoc or Šidák’s test. ***P ≤ 0.001, compared with sham-operated animals; ^##^*P* ≤ 0.01, clinical-like compared with prodromal-like; ^#^*P* < 0.05; ^§§§^*P* ≤ 0.001, before surgery compared with after surgery.

Although PD diagnosis in humans relies on a scale that integrates evaluations of both neuropsychiatric and primarily motor symptoms (24), its final confirmation relies on postmortem histological evaluations that include dopaminergic loss in the nigrostriatal pathway, leading to denervation of the DS, and the presence of α-synuclein containing Lewy bodies. In order to categorize each 6-OHDA rat presenting its own PD phenotype without temporal evolution of its symptoms and its dopaminergic lesions in a subgroup corresponding to a specific disease stage (i.e., prodromal-like or clinical-like), we established a score, called the Parkinson’s disease progression (PDP) score. The PDP score was based on the same kind of criteria used for patients, i.e., (a) the neuropsychiatric component, evaluated by operant self-administration performances (motivation); (b) fine motor capacities, evaluated by stepping test performances; (c) and extent of the DS lesion, evaluated by postmortem histological analysis. Based on this score, we assigned the PD animals to 2 categories, prodromal-like or clinical-like. These terms will be used throughout the study (Figure 2A).

Tyrosine hydroxylase immunoreactivity (TH-IR) revealed that bilateral 6-OHDA injection in the substantia nigra pars compacta (SNc) led to a partial nigrostriatal dopaminergic lesion resulting in dopaminergic denervation in the DS and, to a lesser extent, in the Nacc (Figure 2, B and C). Indeed, TH-IR quantification showed a significant loss (62.2%; *P* ≤ 0.001) of dopaminergic projections in the DS of prodromal-like animals compared with sham-operated animals, with a greater loss in clinical-like animals (71.96%). A slight loss of TH-IR was also observed in the Nacc of 6-OHDA animals (Figure 2C). This denervation pattern preserves learning and global ambulatory activity of animals (19), allowing study of motivational processes without the potential bias related to locomotor alterations often present in PD animal models (refs. 19, 22, and Supplemental Figure 1; supplemental material available online with this article; https://doi.org/10.1172/JCI146400DS1). Motivation was measured by a sucrose self-administration procedure. Prior to surgery, rats learned the motivation task and reached their maximum performance level (about 80 rewards per 1-hour session). After surgery, the performances of sham-operated rats remained stable, while those of 6-OHDA animals dramatically decreased (30%–66%; Figure 2D). Regarding fine motor skills, the 6-OHDA infusion did not reduce the number of adjusting steps, except in the clinical-like group, where it strongly decreased (Figure 2E). We then investigated the metabolic dysregulations of each animal in association with its PDP score, i.e., its PD stage.

### Serum metabolic signature coevolves with PD progression in 6-OHDA rats.

**Figure 3:**
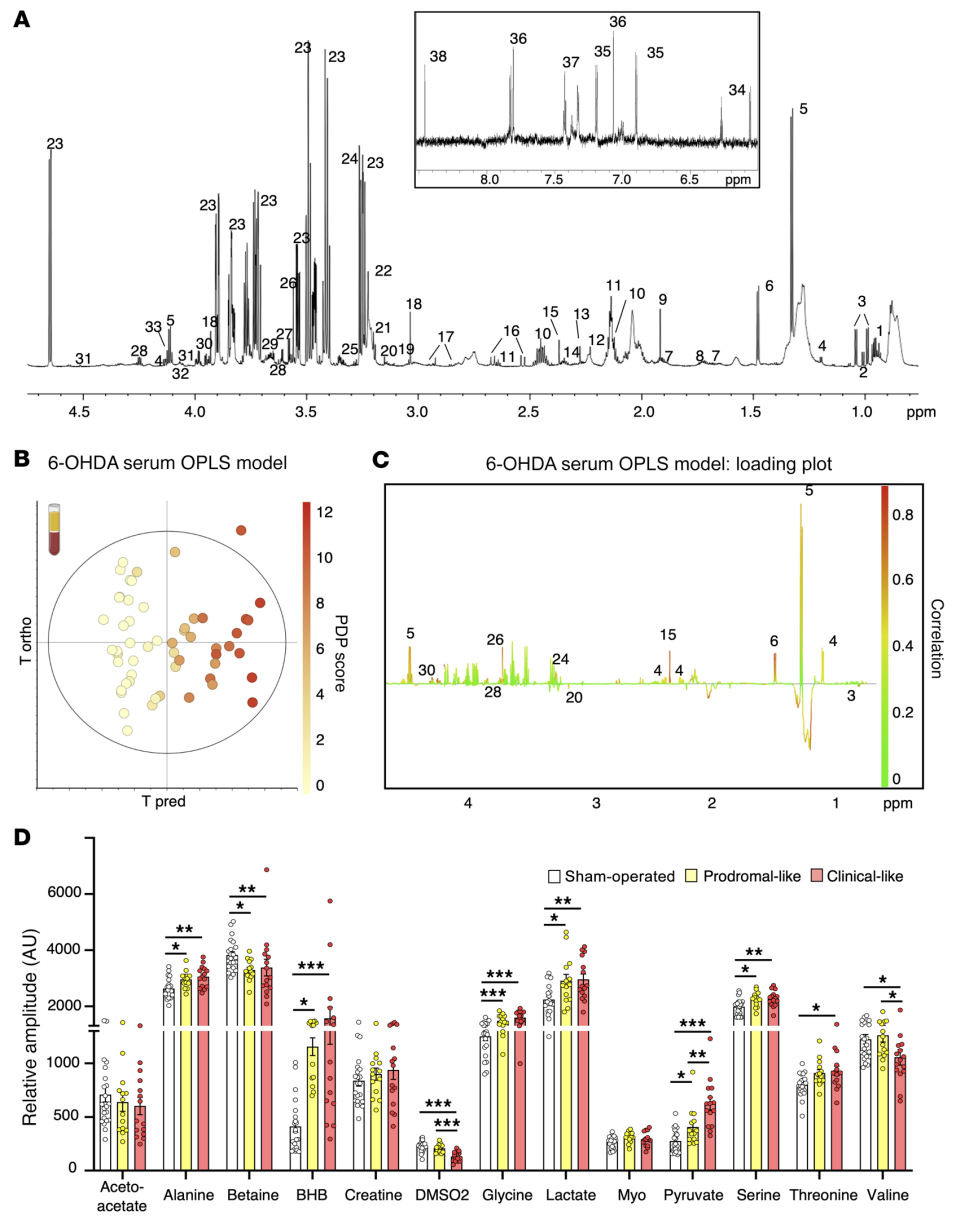
Serum metabolic profile of 6-OHDA rats evolves with PD progression. (**A**) Example of ^1^HNMR spectrum at 950 MHz (δ0.5–4.7 ppm and δ6–8.5 ppm) using CPMG pulse sequence. Assignment was as follows: 1, isoleucine; 2, leucine; 3, valine; 4, BHB; 5, lactate; 6, alanine; 7, arginine; 8, lysine; 9, acetate; 10, glutamine; 11, methionine; 12, acetone; 13, acetoacetate; 14, glutamate; 15, pyruvate; 16, citrate; 17, asparagine; 18, creatine; 19, phosphocreatine; 20, DMSO2; 21, choline; 22, PC; 23, glucose; 24, betaine; 25, myoinositol; 26, glycine; 27, glycerol; 28, threonine; 29, glycerophosphocholine; 30, serine; 31, ascorbate; 32, glycerate; 33, proline; 34, deoxycytidine triphosphate; 35, tyrosine; 36, histidine; 37-phenylalanine; 38, formate. Macromolecules are not specified (see Supplemental Table 2). (**B** and **C**) OPLS model built with ^1^HNMR spectra of serum samples from 6-OHDA (*n* = 29) and sham-operated (*n* = 22) rats and their PDP scores: the 6-OHDA serum OPLS model. *R2Y* = 0.926; *Q2* = 0.604; 1 predictive and 3 orthogonal components; CV-ANOVA, *P* = 3.47 × 10^–7^. (**B**) Score plot versus the first predictive and first orthogonal components. A clear gradation of color is observed from left to right, showing that metabolic profiles evolve with PD progression. (**C**) Loadings plotted in 1D with NMR variables color coded for their correlation with PD score from green (low correlation) to red (high correlation). Positive peaks indicate upregulated metabolites along with increasing PDP score, while negative peaks indicate downregulated metabolites along with PDP score evolution. (**D**) Relative amplitude of the metabolites most involved in metabolic gradation in 6-OHDA animals, i.e., alanine, betaine, BHB, DMSO2, glycine, lactate, pyruvate, serine, threonine, and valine in sham-operated (*n* = 22), prodromal-like (*n* = 14), and clinical-like (*n* = 15) animals. Data are represented as mean ± SEM, 1-way ANOVA followed by Tukey’s post hoc test and correction for multiple comparisons. *P ≤ 0.05; **P ≤ 0.01; ***P ≤ 0.001.

Figure 3A illustrates a typical proton nuclear magnetic resonance (^1^H NMR) spectrum of 6-OHDA rat serum, acquired at an ultrahigh magnetic field (23T). This allowed the identification of approximately 50 metabolites (Supplemental Table 2). The score plot of the orthogonal partial least squares (OPLS) analysis, performed with the NMR data and PDP scores for each animal (i.e., the 6-OHDA serum OPLS model) shows a clear gradation of dot colors from “cold” (light yellow) on the left to “hot” (red) on the right. This indicates that the metabolic profile coevolves with disease progression (Figure 3B).

The animals were assigned to different disease-stage groups afterwards, according to their individual PDP scores, in order to mimic a clinical approach (see Methods). Among the 14 metabolites with a correlation with PDP score of 0.5 or more in the OPLS (Figure 3C), we found that 10 were modified in at least 1 PD-like group compared with those in sham-operated animals (Figure 3D): alanine, betaine, β-hydroxybutyrate (BHB), dimethyl sulfone (DMSO2), glycine, lactate, pyruvate, threonine, serine, and valine. These metabolites did not evolve identically with disease progression. First, 6 metabolites were modified in the prodromal-like stage: alanine, BHB, glycine, lactate, and serine increased, while betaine decreased. Their levels then remained stable in the clinical-like stage. Secondly, pyruvate progressively increased as PD progressed. Finally, 3 metabolites were only modified at the clinical-like stage: DMSO2 and valine decreased, while threonine increased (Figure 3D). Thus, metabolic dysregulations observed in the sera of 6-OHDA rats could be highly predictive of each PD-like stage.

### Brain tissue metabolic profiles in the 6-OHDA rat model reflect dysregulations in serum.

**Figure 4:**
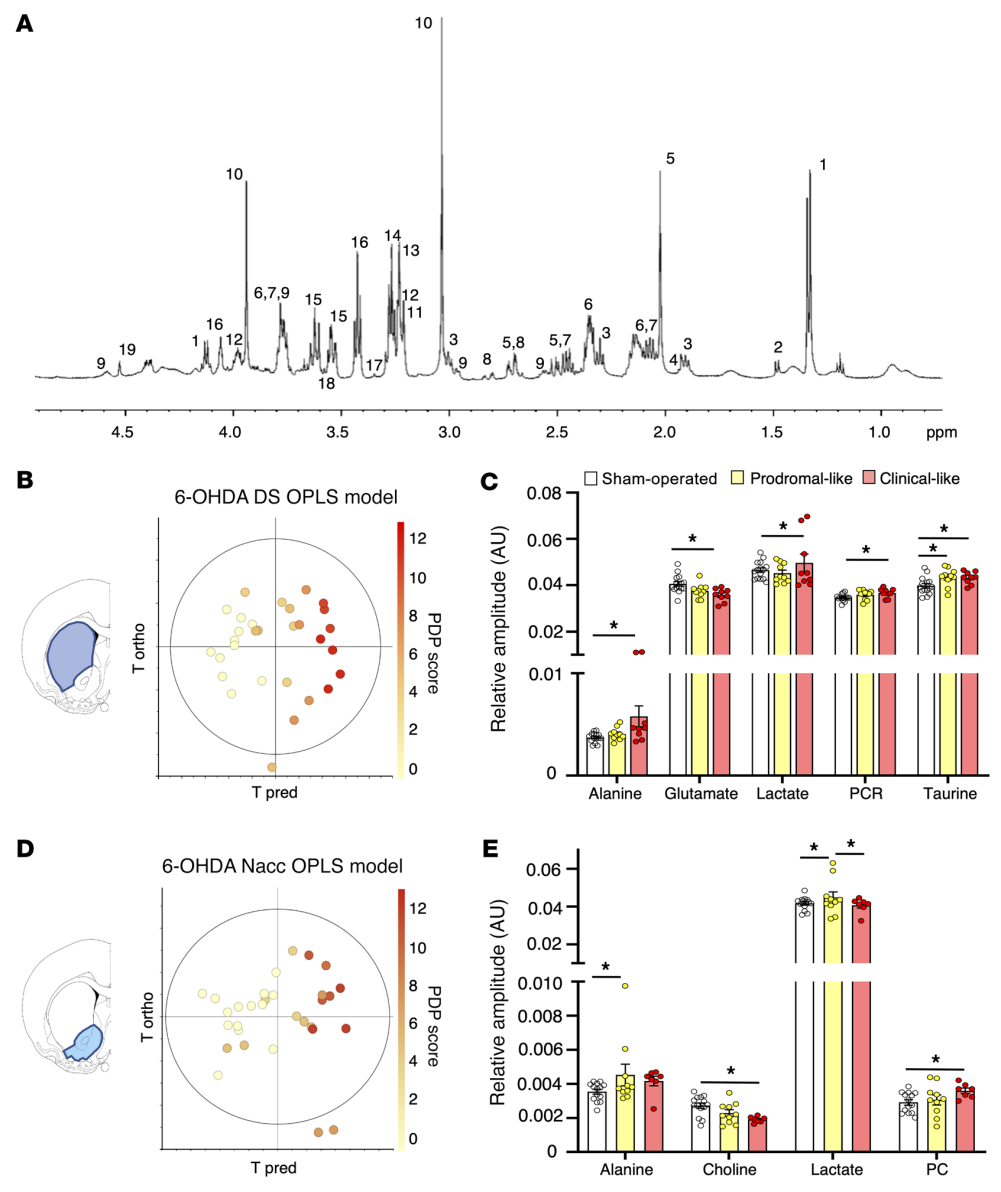
Brain metabolic profile of 6-OHDA rats is gradually modified during PD progression, consistently with serum results. (**A**) Example of ^1^H HRMAS NMR spectra of 6-OHDA rat rain (DS) at 500 MHz. Assignment was as follows: 1, lactate; 2, alanine; 3, GABA; 4, Acetate; 5, N, acetylaspartate; 6, glutamate; 7, glutamine; 8, aspartate; 9, gluthation; 10, phosphocreatine and creatine; 11, choline; 12, phosphoethanolamine; 13, PC; 14, glycerophosphocholine; 15, myoinositol; 16, taurine; 17, scyllo-inositol; 18, glycine; 19, ascorbate. (**B**) Score plot of the OPLS model built with ^1^H HRMAS NMR spectra of DS versus the first predictive and the first orthogonal components (6-OHDA DS OPLS model). There is clear color gradation from left to right, showing that metabolic profiles evolve with PD progression. *R2Y* = 0.883; *Q2* = 0.703, 1 predictive and 3 orthogonal components, CV ANOVA *P* = 0.0003. (**C**) Relative amplitude of metabolites in DS in sham-operated (*n* = 15), prodromal-like (*n* = 10), and clinical-like (*n* = 9) 6-OHDA animals for the 5 key metabolites implicated in metabolic gradation observed in the OPLS, i.e., alanine, glutamate, lactate, phosphocreatine, creatine (PCR), and taurine. (**D**) Score plot of the OPLS models built with ^1^H HRMAS NMR spectra, of Nacc versus the first predictive and first orthogonal components (6-OHDA Nacc OPLS model). Color gradation from left to right shows the evolution of metabolic profiles with PD progression. *R2Y* = 0.690; *Q^2^* = 0.523, 1 predictive and 1 orthogonal component, CV ANOVA, *P* = 0.0005. (**E**) Relative amplitude of metabolites in Nacc in sham-operated (*n* = 14), prodromal-like (*n* = 10), and clinical-like (*n* = 7) 6-OHDA animals for the 4 key metabolites implicated in metabolic gradation observed in the OPLS, i.e., alanine, choline, lactate, and PC. Data are represented as mean ± SEM, 1-way ANOVA followed by Tukey’s post hoc test and correction for multiple comparisons. *P ≤ 0.05.

Proton high-resolution magic angle spinning (^1^H HRMAS) NMR spectroscopy was used to investigate the metabolic profiles of unprocessed brain biopsies (DS and Nacc) from 6-OHDA animals, sampled at the same levels as the sections used for histology to quantify dopaminergic denervation. A representative spectrum of a DS biopsy from a 6-OHDA rat is shown in Figure 4A, with labeling of assigned and quantified metabolites. Regarding DS samples, the OPLS analysis (i.e., the 6-OHDA DS OPLS model) revealed that cerebral metabolic profiles coevolved with individual PDP scores, as in serum (Figure 4B). The significant dysregulations of key metabolites are illustrated in Figure 4C. Compared with those in sham-operated animals, one metabolite, taurine, was significantly increased in the prodromal-like group (*P* ≤ 0.05), while 4 metabolites were significantly modified in the clinical-like group: alanine, lactate, phosphocreatine and creatine (PCR) increased, and glutamate decreased.

The OPLS analysis of Nacc data (i.e., the 6-OHDA Nacc OPLS model) showed the same trend, although less clearly (Figure 4D). The univariate statistics revealed a substantial increase of alanine and lactate in the prodromal-like group, whereas the clinical-like group was characterized by an increase of phosphocholine (PC) and a decrease of choline compared with that in sham-operated animals (Figure 4E). These experiments show that both alanine and lactate exhibit similar dysregulation between serum and brain.

### Pramipexole partially reverses serum and tissue metabolic dysregulations induced by 6-OHDA.

**Figure 5:**
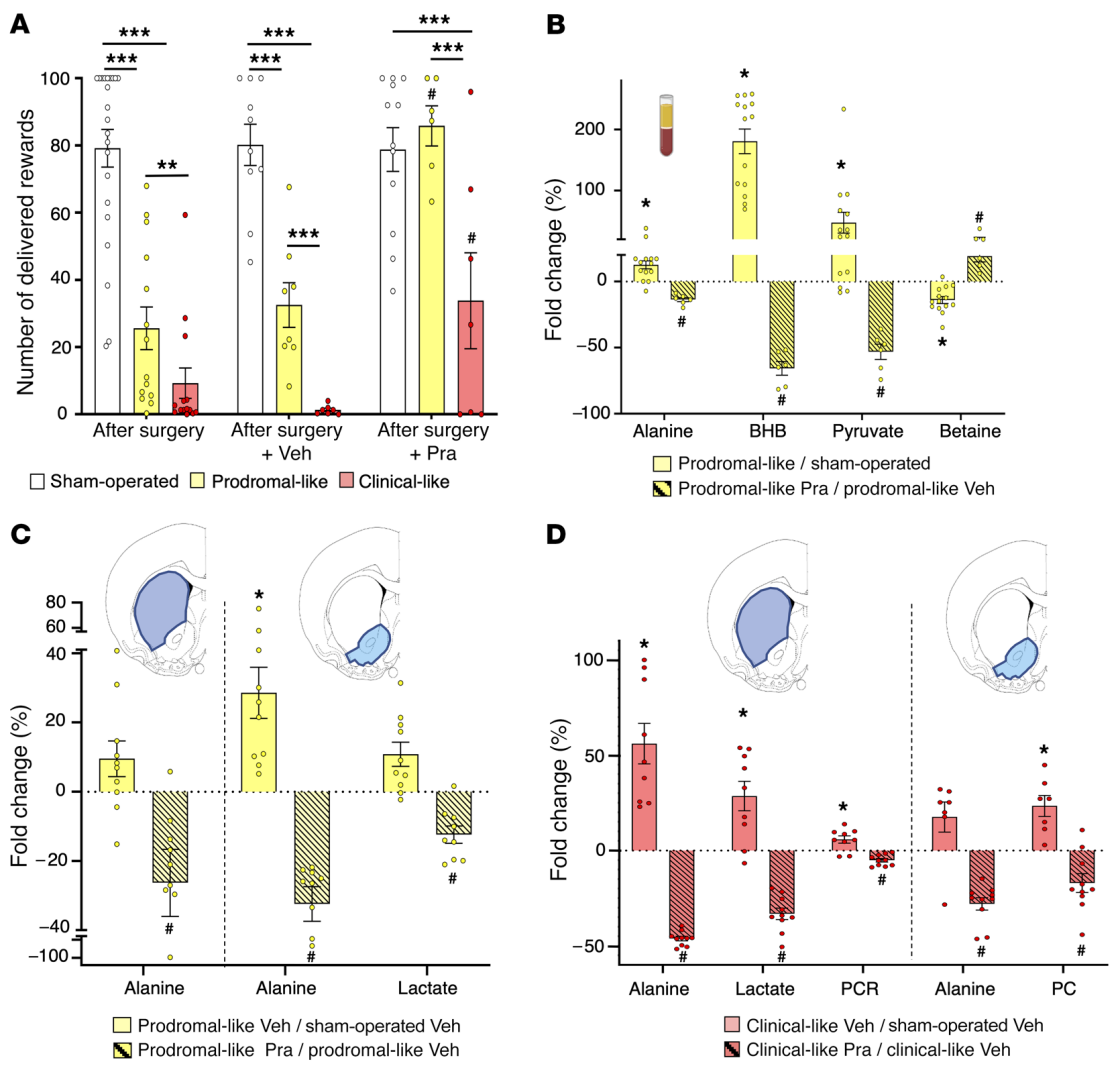
Impact of chronic treatment with a dopaminergic agonist (Pra) on behavioral and metabolic dysregulations of 6-OHDA rats. (**A**) Self-administration performances. Mean number of sucrose deliveries per session after surgery or after chronic administration of Veh or Pra (last 3 days). Pra treatment (*n* = 6) induced a total reversion of the lesion effect on the number of sucrose deliveries in prodromal-like compared with Veh (*n* = 8). In clinical-like animals, Pra treatment (*n* = 7) induced a moderate increase in the number of sucrose deliveries compared with Veh (*n* = 7) without reaching the performance of sham-operated animals (*n* = 12). (**B**) Serum metabolic dysregulations. Percentage of variation of serum metabolites between control and prodromal-like animals (*n* = 14; open bar) and between prodromal-like animals treated with Veh or Pra (*n* = 6; hatched bar). Alanine, betaine, BHB, and pyruvate vary in the opposite direction compared with the lesion effect. (**C** and **D**) Brain metabolic dysregulations. Percentage of variation of brain tissue metabolites normalized to sham-operated animals treated with Veh in DS and in Nacc. Note in prodromal-like (**C**) and clinical-like animals (**D**), respectively, the reversion of metabolic dysregulation induced by Pra (*n* = 9 for DS and *n* = 10 for Nacc for both groups; hatched bar) compared with Veh (*n* = 10 for each structure in prodromal-like animal; *n* = 9 for DS and *n* = 7 for Nacc in clinical-like animal; filled bar). Data are represented as mean ± SEM, 1-way ANOVA followed by Tukey’s post hoc test. *P ≤ 0.05; **P ≤ 0.01; ***P ≤ 0.001; ^#^*P* ≤ 0.05, prodromal-like or clinical-like Pra compared with prodromal-like or clinical-like Veh.

We further evaluated whether the serum metabolic profile could be influenced by the use of pramipexole (Pra), a widely used dopaminergic treatment in newly diagnosed PD patients that is known to improve only neuropsychiatric symptoms at low doses in animal models (22, 25). As expected, after 15 days of subchronic administration of Pra, the deficits in the self-administration task measured in 6-OHDA rats were fully and partially reversed in prodromal-like and clinical-like animals, respectively. In contrast, the performances of rats treated with vehicle (Veh) did not improve (Figure 5A).

Concerning the serum metabolic profiles, to simultaneously visualize the effect of the 6-OHDA lesion and the treatment, the metabolite levels of prodromal-like animals were normalized to their levels in sham-operated animals (lesion effect) or in lesioned animals given Veh (Pra effect). In prodromal-like animals, the alanine, BHB, and pyruvate levels increased after lesion (Figure 3D and Figure 5B) and were significantly decreased by Pra (Figure 5B), while betaine decreased after lesion, but increased in Pra animals. In the clinical-like group, metabolic reversion induced by Pra was very limited and concerned only alanine. This is consistent with the modest behavioral reversion observed (slight effect on the self-administration task and no effect on the motor task; ref. 25, data not shown).

In contrast to serum, for which several longitudinal samples could be taken in the same animal, brain samples could only be collected at the end of the experiment, when all animals had received chronic Pra treatment. Therefore, sham-operated animals treated with Veh were used to normalize the metabolite levels. As in serum, Pra administration caused a decrease of some metabolites previously increased by 6-OHDA lesions. In prodromal-like animals, this was observed for alanine in DS and Nacc and for lactate in Nacc (Figure 5C). In the clinical-like group, the alanine levels after Pra evolved similarly to those of prodromal-like animals, and lactate was significantly modified in DS. Furthermore, we observed a reversion of PCR and PC levels in DS and Nacc respectively (Figure 5D).

Overall, the 6-OHDA rat model showed substantial alterations in both the serum and tissue metabolomes, associated with the progression of the disease and already detectable in the prodromal-like stage. Moreover, some of these alterations were partially reversed by chronic administration of Pra, which also reversed neuropsychiatric deficits. In order to assess the specificity of the biomarkers found in the 6-OHDA model regarding progression and pathophysiological mechanisms of the disease, we extended the serum metabolomics study to 2 other PD animal models.

### 6-OHDA rats, α-synuclein rats, and MPTP monkeys share common serum metabolic perturbations.

First, using the same rat strain (Sprague-Dawley) as in the 6-OHDA study, we selected an α-synuclein rat model in which the overexpression of human A53T α-synuclein was induced in the SNc using adeno-associated viral vectors (AAV). This expression leads to progressive neurodegeneration in the targeted region, allowing follow-up of the different stages of PD in the same animal (20), namely, a prodromal-like stage approximately 3 weeks after injection and a clinical-like stage after approximately 10 weeks, characterized by degeneration of the nigrostriatal pathway accompanied by motor dysfunctions reminiscent of the impairments observed in the clinical phase of PD (Supplemental Figure 2).

**Figure 6:**
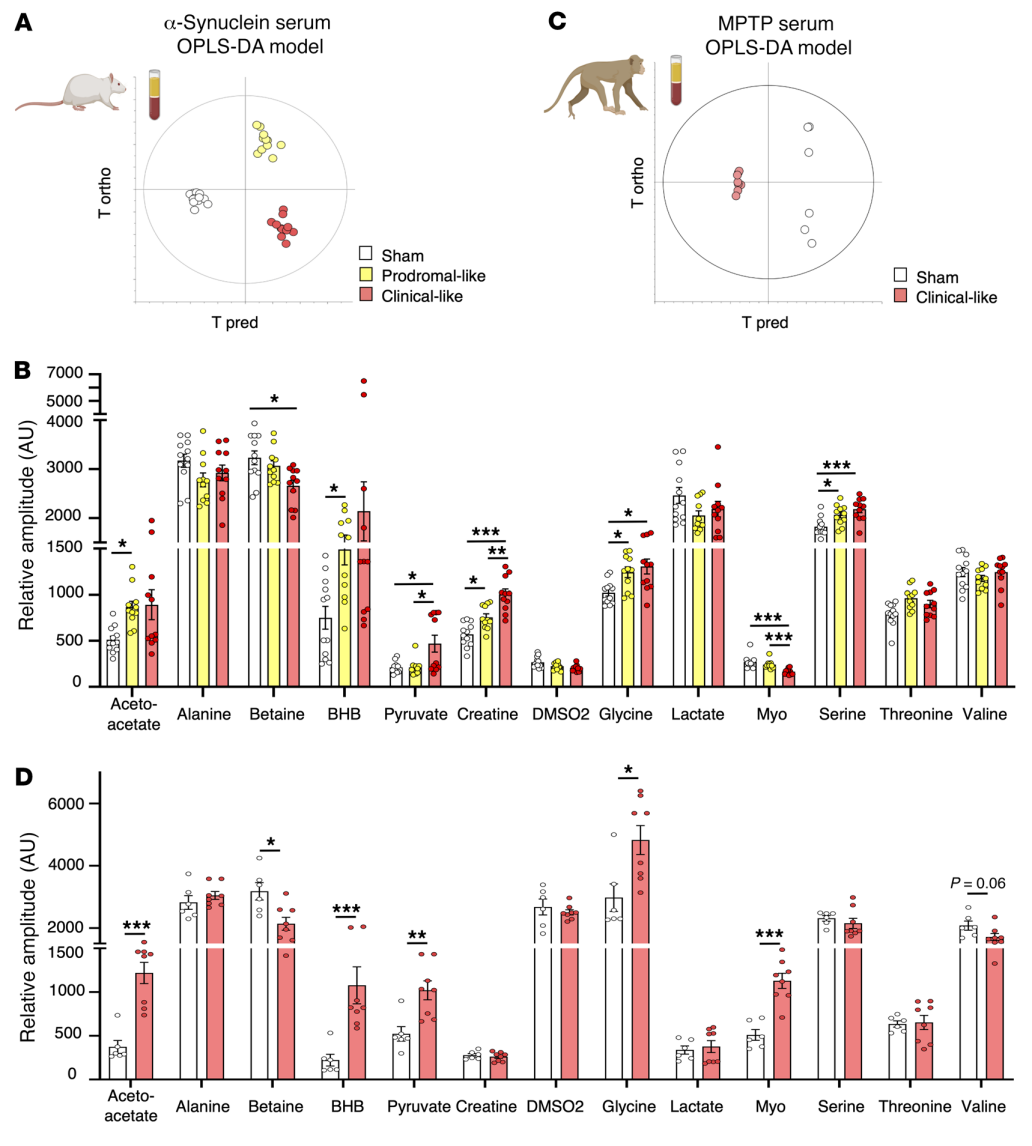
Alternative PD models from different animal species show similar metabolic dysregulations in serum. (**A**) Score plot of the OPLS-DA model built with ^1^HNMR spectra of serum samples from α-synuclein rats versus first predictive and first orthogonal components (α-synuclein OPLS model). Three groups are clearly discriminated, mimicking 3 different stages of PD (sham operated, *n* = 12; prodomal-like, *n* = 11 clinical-like *n* = 11). *R2Y* = 0.929; *Q2* = 0.518; 1 predictive and 3 orthogonal components; CV ANOVA, *P* = 0.008. (**B**) Relative amplitude of most discriminating metabolites in the α-synuclein OPLS model, i.e., acetoacetate, betaine, BHB, creatine, glycine, myoinositol (myo), pyruvate,and serine in sham-operated (*n* = 12), prodromal-like (*n* = 11), and clinical-like (*n* = 11) animals. (**C**) Score plot of the OPLS-DA model built with ^1^HNMR spectra of serum samples from nonhuman MPTP primates versus first predictive and first orthogonal components (MPTP OPLS-DA model). Sham-operated (*n* = 6) and clinical-like (*n* = 8) animals are clearly discriminated. *R2Y* = 0.998; *Q^2^* = 0.963; 1 predictive and 1 orthogonal component; CV ANOVA *P* = 0.0005. (**D**) Relative amplitude of most discriminating metabolites in the MPTP OPLS-DA model, i.e., acetoacetate, alanine, betaine, BHB, creatine, lactate, pyruvate, and valine in sham-operated (*n* = 6) and MPTP (*n* = 8) animals. Data are represented as mean ± SEM, mixed model followed by Tukey’s post hoc test or Mann-Whitney test. *P ≤ 0.05; **P ≤ 0.01; ***P ≤ 0.001.

OPLS-DA analysis was used to evaluate whether a specific metabolic signature was associated with each disease stage (i.e., the α-synuclein serum OPLS-DA model). Sham-operated animals (i.e., infected with GFP) exhibited no difference among the 3 time points, 0, 3, and 10 weeks after injection (Supplemental Figure 3), while for α-synuclein animals, the 3 time points were clearly separated (Figure 6A), indicating that each stage was characterized by a specific metabolic signature. In particular, an increase of BHB, glycine, pyruvate, and serine and a decrease of betaine were observed over time, as in 6-OHDA rats. Moreover, in the α-synuclein rat model only, myo-inositol decreased, while acetoacetate and creatine increased (Figure 6B).

These results revealed similarities in metabolic disturbances for 2 different PD rat models that were associated with disease progression. To further investigate, we performed metabolic analysis in a nonhuman primate PD model, the MPTP model, which mimics the clinical stage of PD and presents a greater homology with humans (26).

The OPLS-DA model presented in Figure 6C (i.e., the MPTP serum OPLS-DA model) showed clear discrimination between sham-operated and MPTP groups. On the one hand, nonhuman MPTP primates presented a significant increase of lactate and a decrease of valine, as in 6-OHDA rats. On the other hand, acetoacetate and creatine significantly increased, as in the α-synuclein rats. Finally, we observed an increase of BHB and pyruvate and a decrease of betaine, as in both rodent models (Figure 6D). Taken together, these results reveal common metabolic alterations associated with PD progression in 3 different animal models performed in 2 different species. To further validate the clinical relevance of these markers, we compared these preclinical results to those obtained in PD patient samples.

### Serum metabolic signatures of de novo PD patients and PD animal models have similarities.

**Figure 7:**
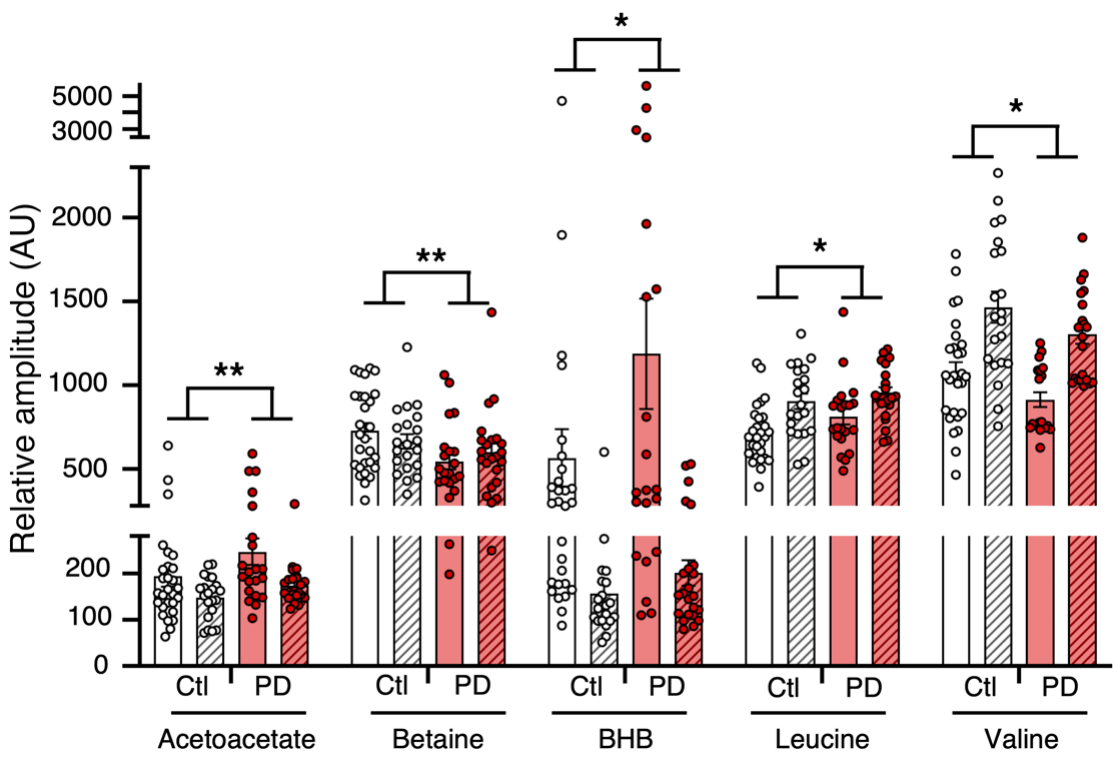
Main metabolic changes between PD patients and controls in NIH and Italian cohorts. For each cohort, a *t* test was performed to find significant differences between PD patients (*n* = 19 for NIH and *n* = 23 for first Italian cohorts) and healthy control subjects (*n* = 30 for NIH and *n* = 21 first Italian cohorts), while 2-way ANOVA followed by Šidák’s post hoc test was performed on both cohorts grouped to highlight pathology effects. *P ≤ 0.05; **P ≤ 0.01.

Each cohort was first analyzed individually, i.e., each de novo PD patient group (*n* = 19/21 NIH/Italy) was compared with an age- and sex-matched control group (*n* = 30/23 NIH/Italy). Except for some metabolites, such as alanine (data not shown), the observed variations were consistent between the 2 cohorts, as shown in Figure 7. For instance, we observed a marginal increase of BHB and acetoacetate in Italian PD patients (+28.3%, *P* = 0.24 and +17.2%, *P* = 0.05, respectively), which was even higher in NIH PD patients (+103.2%, *P* = 0.09 and +28.1%, *P* = 0.12).

To increase statistical power, the 2 cohorts were pooled (*n* = 42 PD, *n* = 53 control). Nevertheless, as the data variance was mostly affected by sample origin, i.e., Italian or NIH, independently of the case (PD or control), we performed a 2-way ANOVA with (a) the origin of the cohort and (b) the case (control or PD) as factors to extract PD information only. Figure 7 presents the metabolites significant for the case factor only, without interaction between the cohort origin and the case. The increase of BHB and acetoacetate observed in each cohort became significant when pooled. Betaine was significantly decreased in the serum of PD patients, as in the α-synuclein and MPTP models. Moreover, valine significantly decreased, as observed in the 6-OHDA rat model. Finally, a trend toward a decrease of leucine was noted. Moreover, the analysis of a subset of serum samples from other de novo PD patients treated with 0.5 to 4.5 mg/d Pra (*n* = 9) revealed a tendency toward normalization of BHB and betaine levels compared with those of nontreated PD patients, as observed in 6-OHDA rats (Supplemental Figure 4).

### Serum metabolites as putative composite biomarker for early diagnosis of PD.

We next assessed the diagnostic value of the metabolites found to discriminate prodromal-like animals from sham operated or de novo PD patients from controls. We developed multiple logistic regression models based on the metabolites that were statistically significant in the above comparisons and in at least 3 of the 4 PD models studied (6-OHDA, α-synuclein, MPTP, and human), i.e., acetoacetate, betaine, creatine, BHB, pyruvate, and valine. We tested each metabolite individually and all possible combinations thereof and pooled prodromal-like animals from the 6-OHDA and α-synuclein models.

**Figure 8:**
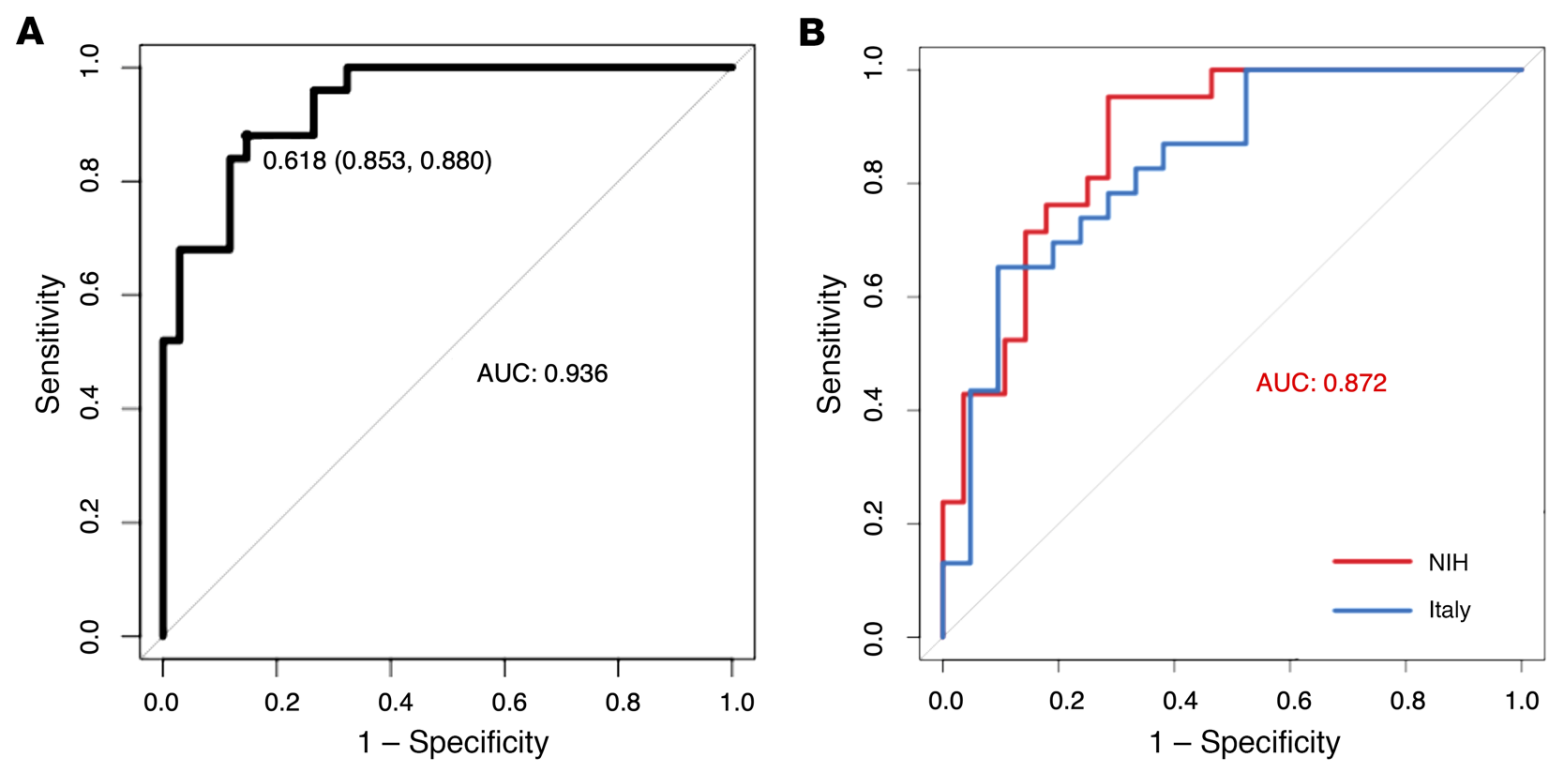
Logistic regression provided a serum biomarker composed of 6 metabolites: BHB, acetoacetate, valine, creatine, betaine, and pyruvate. (**A**) ROC curve with serum samples of prodromal-like (*n* = 25) and sham-operated (*n* = 34) animal models (6-OHDA and α-synuclein rats) (AUC = 0.936; sensitivity: 0.853; specificity: 0.88). (**B**) ROC curve with data of PD patients and matched controls of NIH cohort in red (*n* = 19 and *n* = 30; AUC = 0.88; sensitivity: 0.957; specificity: 0.714) and first Italian cohort in blue (*n* = 23 and *n* = 21; AUC = 0.83; sensitivity: 0.65, specificity: 0.90).

The best regression model, as evaluated by the receiver operating characteristic (ROC) curves, retained all 6 metabolites, using a regression based on the following predictive algorithm: (*P* = 1.107 × 10^–15^): logit(*P*) = log(*P*/(*1* − *P*)) = –54.72 + 0.49 BHB + 0.39 pyruvate – 0.42 valine + 0.06 acetoacetate + 0. 52 creatine – 0.10 betaine. The ROC curve had an AUC of 0.936. By applying the optimal threshold of 0.618 (sensitivity, 0.853, specificity, 0.880; Figure 8A), we found an accuracy of 92.5%, indicating that 37 of the 40 animals included in the validation cohort (clinical-like rats and all primates) were correctly predicted regarding what group they belonged to.

Concerning human samples, we used the NIH cohort to train the model since (a) this was the largest cohort and (b) it included only recently diagnosed patients (de novo patients diagnosed ≤ 1 year) in contrast with the Italian cohort, which included patients with PD duration of 3 years or less, defined from symptom onset to diagnosis. Again, as with animal data, the best regression model retained all 6 metabolites and was based on the following predictive algorithm: (*P* = 1.95 × 10^–4^): logit(*P*) = log(*P*/(1 − *P*)) = −42.78 + 0.16 BHB – 3.31 pyruvate – 12.01 valine + 7.06 acetoacetate + 3.14 creatine – 7.79 betaine. The subsequent ROC curve had an AUC of 0.87. The optimum threshold was 0.279, and corresponding sensitivity and specificity were, respectively, 0.952 and 0.714 (Figure 8B). Using this classifier, the subjects of the Italian cohort were predicted to have PD with 82.6% accuracy, with 17.4% false negatives. Consistently, the ROC curve built with the same classifier and Italian cohort had an AUC of 0.83 (See Figure 8B). To perform a first prospective-like validation, samples from a second set of de novo PD patients from Italy (*n* = 36), obtained after all the above-described analyses, were submitted to the same NMR-based metabolomics workflow, and 84.6% of them were classified as PD.

Finally, a multiple logistic regression was performed using serum data from all PD animals (prodromal-like and clinical-like) from all models using the same 6 metabolites and was evaluated with the subsequent ROC curve presenting an AUC of 0.954 (Supplemental Figure 5) and an accuracy of 88.9%. Altogether, the accuracy of the composite biomarker developed from the same 6 serum metabolites was at least 82.6% whatever the species.

## Discussion

Late and inaccurate diagnosis of PD (3, 27) occurs if patient management and therapies are too restricted. Therapies remain only symptomatic and become ineffective after several years. Although many molecules have been tested, there are still no agents or neuroprotective therapies to efficiently slow down, stop, or reverse neurodegeneration in PD patients, despite promising theoretical or preclinical evidence, partly because treatment is applied too late (28–30). Thus, finding easily measurable and highly predictive biomarkers of the prodromal phase of the disease appears as a milestone for the acceleration of curative therapeutic development and improvement of patient care.

In the present study, using NMR-based metabolomic approaches, we observed alterations of the metabolome in serum and tissue of different animal models mimicking the different stages of PD, including the prodromal phase. NMR provides highly reproducible results and requires minimal sample preparation, which is compatible with multicentric studies and clinical routines. Concerning tissue samples, despite the absence of some metabolites, such as pyruvate and citrate, mainly due to postmortem effects (31), we were able to confirm dysregulation observed in serum, demonstrating that blood could reflect central dysfunction (32), at least partially. In addition, the present multimodel approach overcame the intrinsic limitations of each animal model and increased the incomplete predictive value of each model alone, revealing a characteristic set of dysregulated metabolites as a potential biomarker. Finally, we extended the study to human serum samples from 2 biobanks to validate the clinical relevance of our biomarker of PD.

We have demonstrated that the profiles of 6 metabolites, acetoacetate, betaine, BHB, creatine, pyruvate, and valine, combined together, could constitute an accurate PD composite biomarker for animal models and for human samples. The use of this biomarker allowed us to discriminate NIH de novo PD patients from healthy controls (AUC = 0.88) and Italian de novo PD patients from healthy controls (first cohort), though to a slightly lesser degree (AUC = 0.83). The divergent preanalytical procedure applied for serum sampling in the 2 cohorts, (i.e., tubes used, sample processing, etc.), may represent a major source of experimental variability that could explain this difference (33, 34). Moreover, it should be noted that the human samples were collected under a standardized experimental protocol that was very strict, but not metabolomics designed. This reflects the power of metabolomics and the robustness of our results and supports the view that serum biomarkers, easier to use and less invasive than imaging methods, present a similar efficiency (35). Moreover, in an analysis one year later, 84.6% of the additional de novo PD patients (second Italian cohort) were classified as having PD, which clearly demonstrates the robustness of NMR-based metabolomics and provides an initial prospective-like validation of the composite biomarker.

Interestingly, the dysregulation of 3 of the 6 metabolites composing the biomarker, i.e., betaine, BHB, and pyruvate, was partially corrected by chronic treatment with Pra in prodromal-like 6-OHDA rats as well as their motivational deficits. This partial pharmacological reversion was also observed in a subset of de novo patients for betaine and BHB. Pra, classically used in PD patients to improve symptoms and as a dopaminergic agonist, could result in changes in dopaminergic transmission, the alteration of which represents a major hallmark of PD pathophysiology. Therefore, this partial pharmacological reversion observed both in animals and in de novo patients reinforces the specificity of our biomarker regarding changes in dopaminergic transmission, which is relevant to PD pathophysiology and demonstrates the power of metabolomics for identifying robust biomarkers, potentially monitoring therapeutic outcomes, in a less invasive and more convenient way than the current methods, such as the DaTscan (36).

**Figure 9:**
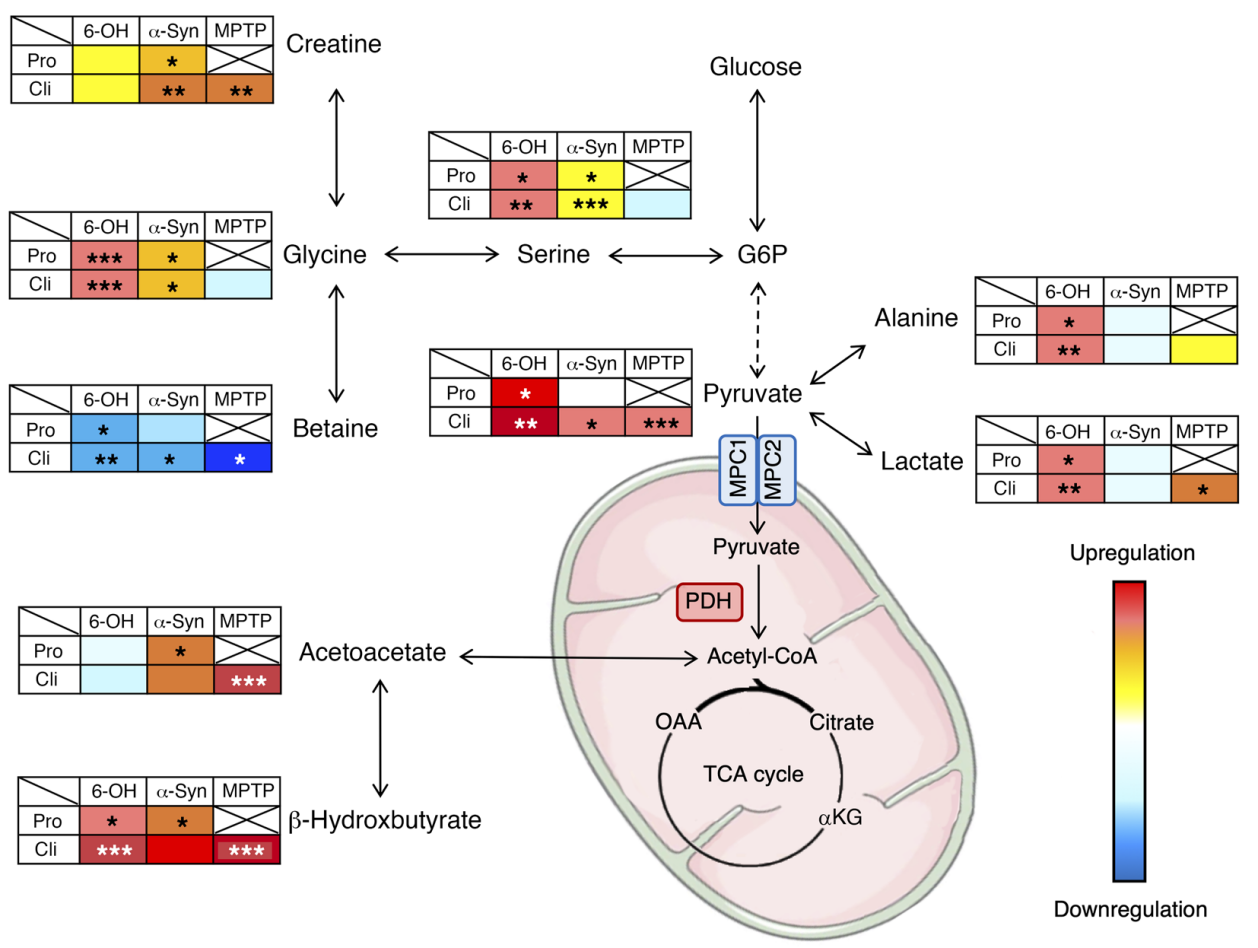
Schematic representation of altered metabolic pathways in different PD animal models mimicking different stages of the disease, suggesting possible reprogramming of pyruvate metabolic pathway. Degrees of dysregulation were represented by color gradation compared with sham-operated animals in each model. *P < 0.05; **P < 0.01; ***P < 0.001. Pro, prodromal-like; Cli, clinical-like; G6P, glucose-6-phosphate; OAA, oxaloacetate; αKG: α- ketoglutarate; α-syn, α-synuclein.

The metabolic dysregulations reported in the present study could provide some clues to the mechanisms impacted during PD progression. In the 6-OHDA model, the gradation of metabolic profiles was clearly associated with the evolution of the PDP score even from very early on. In particular, glycolytic (e.g., pyruvate) and associated metabolites (e.g., lactate) were increased. Interestingly, these increases were also observed in the α-synuclein and primate models, except for lactate in α-synuclein rats (Figure 9). Importantly, lactate increase was also observed in DS and Nacc and may reflect upregulation of the activity of the astrocyte-neuron lactate shuttle (37, 38), known to play a major role in central nervous system homeostasis and energy metabolism (39, 40), especially in energy production. It is noteworthy that alanine, intrinsically linked to this shuttle (40), was also increased in sera and tissues of 6-OHDA animals.

Serine and glycine, possibly deriving from the first steps of glycolysis (41), were increased in the 2 rat models studied. They have been described as influencing mitochondrial dynamics and homeostasis (42) and are, furthermore, coagonists of NMDA receptors (43). Their increase may support glutamatergic hyperactivity, often fundamental to neurodegeneration (44, 45). Betaine and creatine, which can derive from glycine (46, 47), were also dysregulated, but in opposite ways, i.e., betaine was decreased in all animal models, whereas creatine was increased in α-synuclein and primate models. Both may attenuate and protect against oxidative stress (48–51) that represents the primary cause of neuronal death in PD. In the literature, previous metabolomics studies have reported modifications of glycolysis in brain and blood samples of PD animal models (52–54). Dysregulated glycolysis has also been described in animal models expressing neuropsychiatric symptoms similar to those found in the early stages of PD, such as depression or anxiety (55, 56). Together, these observations place glycolysis as a central actor (56, 57) of dysregulations occurring during PD processes and possibly at the first stages of the disease. Surprisingly, in our study, the increase of these glycolytic or glycolytic-linked metabolites (pyruvate, alanine, lactate, glycine, serine) was not associated with a rise in citrate, the first metabolite of the Krebs cycle, also known as tricarboxylic acid (TCA) (other metabolites of the TCA cycle were not observable with the method used). These observations could suggest decoupling of glycolysis from the TCA cycle, leading to an accumulation of glycolytic metabolites. This is in agreement with the increase of ketone bodies (BHB, acetoacetate) in all animal models, which could be used as an alternative fuel to maintain TCA-cycle functioning (Figure 9). Ketone bodies could increase mitochondrial ATP production and support antioxidant defenses (58). Of note, MPTP and 6-OHDA used in animal models are known to exert their cytotoxic activities by depleting mitochondrial ATP in the brain (59). As such, an increase in ATP production in PD mice could protect them from developing motor symptoms (60). Previous studies describing an increase of ketone bodies in blood of PD patients (61) have suggested their possible neuroprotective impact (58, 62, 63). Interestingly, high levels of ketone bodies have been associated with an inhibition of the pyruvate dehydrogenase complex (PDHC) (64), an enzymatic complex located in mitochondria and responsible for the transformation of pyruvate to acetyl-CoA, a crucial step between glycolysis and the TCA cycle. Such inhibition could explain the accumulation of pyruvate and associated metabolites observed in the present study in animal models (65, 66). In human blood, however, a nonsignificant decrease of pyruvate levels was observed, which could account for the negative coefficient found in the regression for pyruvate. This discrepancy between animal models and humans may have different origins, either experimental or metabolic. For instance, metabolic alterations in the blood could only partially reflect, and with some differences between animal models and humans, the metabolic alterations occurring in the brain regions affected in PD. Further investigations on brain tissues will be of great interest to determine exactly how pyruvate metabolism may be implicated in the pathophysiology of PD. However, the downregulation of PDHC gene expression observed in plasma of PD patients (67) as well as a decrease in PDH level in the putamen and SNc (68) are consistent with pyruvate level alteration and with our work. In addition, it has been shown that the mitochondrial pyruvate carrier, which acts an integral part of the shift from glycolysis to the TCA cycle by allowing the transport of pyruvate into the mitochondria, could play an important role in neuronal death and thus represents a possible target for attenuating neurodegeneration (69–71). These 2 intermediaries between glycolysis and the TCA cycle may represent central players in the pathophysiological mechanisms underlying PD and need further investigation.

In summary, our study reports common serum metabolic alterations in 3 animal models mimicking PD, despite coming from different cohorts and species, as early as the prodromal-like phase. Consequently, this common signature is likely to reflect specific alterations linked to PD physiopathology. This provides some clues about the mechanisms involved during PD progression, which now need to be more deeply investigated. In particular, preclinical models displaying a slowly progressive pathology, such as the intrastriatal injection of preformed fibrils of α-synuclein (72) could be useful for further studying the progression of metabolic alterations upon initiation of α-synuclein seeding and for identifying underlying mechanisms. Besides providing clues for deeper investigation of metabolic alterations in animal models, we also found metabolic dysregulations in sera of de novo PD patients (i.e., clinical phase) coming from 2 biobanks not specifically designed for metabolomics. Logistic regression, using the same metabolites as those used for animals, enabled very adequate discrimination between control and PD patients in those 2 cohorts from different geographic origins. A more extended study is now necessary, in particular with prodromal patients.

Furthermore, although our study was performed in PD animal models and PD patients, strongly supporting the specificity of our biomarker for this pathology compared with healthy subjects, we cannot completely exclude that the biomarker may reflect metabolic dysfunctions commonly encountered in neurodegenerative diseases or even more in neurodegenerative disorders with parkinsonian symptoms, such as multiple system atrophy or progressive supranuclear palsy, sometimes mistaken for PD. Thus, with the perspective of clinical application, it would appear necessary to test the ability of our biomarker to discriminate these disorders from PD and thus demonstrate its specificity and potential for the differential diagnosis of parkinsonian syndromes.

Finally, the variations observed in the 3 animal models suggest possible modification in energy metabolism, especially from glycolysis to the TCA cycle. Even if this hypothesis needs additional evidence, we suggest that this alteration may be a crucial point that could be targeted to developing curative PD care.

Our multimodel and translational study demonstrates the usefulness and reproducibility of untargeted metabolomics as a noninvasive approach to searching for biomarkers of PD in animal models. The approach seems particularly promising for use in PD patients, as serum is easily accessible and the biomarker may be relevant even at early stages of the disease when other methods fail.

## Methods

See Supplemental Methods for details regarding surgery, behavioral test, immunochemistry processing and quantification, and NMR experiments.

### Flow chart

Figure 1 illustrates a flow chart of the whole protocol for the animal models. The rats of the first cohort (Figure 1A) were trained for 2 weeks for self-administration until they reached stable performances and were submitted to a stepping test before receiving bilateral intracerebral injection of 6-OHDA (*n* = 29) or NaCl (*n* = 22) in the SNc. After recovery and stabilization of the lesion (around 3 weeks), self-administration was resumed for 1 week, and the stepping test was repeated to monitor the evolution of performances before submitting each group (6-OHDA and NaCl) to 2 weeks of daily injections of 0.2 mg/kg Pra (6-OHDA Pra, *n* = 15; NaCl Pra, *n* = 11) or 0.09% NaCl (6-OHDA NaCl, *n* = 14; NaCl NaCl, *n* = 11). Self-administration was continued throughout Pra treatment, and the stepping test was performed for the last time at the end. Serum samples were collected after surgery, after stabilization of self-administration performances, and at the end of Pra treatment. Brains were collected at the end of the behavioral procedure, snap-frozen in liquid nitrogen, and kept at –80°C before being processed for histology and ^1^H HRMAS NMR experiments. The second cohort of rats (Figure 1B) received intracerebral infusion of AAV-hA53Tα-synuclein (*n* = 12) or AAV-GFP (*n* = 12) in the SNc. Serum samples were taken longitudinally during the study, before and 3 and 10 weeks after AAV infusion.

Monkeys (*n* = 8) received intramuscular MPTP injections (0.3–0.5 mg/kg) every 4 to 5 days over 3 weeks (73). Repeated administration of low MPTP doses was used to mimic a moderate stage, with slow development of the disease and triggering of a moderate dopaminergic lesion. Serum samples were collected at the start of the experiment and after the monkeys had reached a stable parkinsonian state (Figure 1C). All serum samples were analyzed by ^1^H NMR at 950 MHz, and brain samples were submitted to ^1^H HRMAS NMR at 500 MHz.

### Animals

#### Rats.

Experiments were performed on adult male Sprague-Dawley rats (Janvier) weighing approximately 300 g (7 weeks old) at the beginning of the experiment. They were housed under standard laboratory and ethical conditions with reversed light-dark cycle (12 hour light/12-hour dark cycle, with lights on at 7 pm) and with food and water available ad libitum.

#### Monkeys.

Experiments were performed on adult male *Macaca fascicularis* (Tamarinier Ltee, Mauritius Island) weighing between 5 and 8 kg, aged between 3 and 5 years, and housed under standard conditions (12-hour light/12-hour dark cycle; 23°C; 50% humidity).

### Surgery

#### 6-OHDA bilateral injection.

As previously described (19, 22), bilateral injections of 2.3 μL 6-OHDA (3 mg/mL) or NaCl 0.9% (sham) were administered in the SNc (anteroposteriority [AP] = –5.4 mm/lateral [L] = ±1.8 mm/dorsoventral [V] = –8.1 mm).

#### α-Synuclein bilateral injection.

As previously described (20), bilateral injections of AAV-hA53Tα-synuclein (1 μL –7.0 × 10^12^ vg/mL) or AAV-GFP (7.0 × 10^12^ vg/mL, sham-operated animals) were administered in the SNc (AP = –5.1 mm and –5.6 mm/L = ±2.2 mm/V = 8 mm from bregma).

### Behavioral assessment

#### 6-OHDA rats.

Rats were submitted to 2 behavioral tests 3 weeks after surgery when the 6-OHDA lesion was believed to be stabilized (74).

#### Operant self-administration: motivational component.

Rats were trained to self-administer a 2.5% sucrose solution in operating chambers (Med Associates) containing an active, reinforced lever, for which a press resulted in the delivery of 0.2 ml of sucrose solution associated with a light stimulus, and an inactive lever, unreinforced, for which a press caused neither delivery of sucrose nor light stimulus.

#### Stepping test: motor component.

Animals, restrained by the experimenter so that they had 2 hind paws on the ground, were moved over a length of 90 cm by a rectilinear and regular movement from left to right and inversely along a table with a smooth surface. The number of forelimb adjustments during displacement was counted (19).

#### MPTP monkeys.

The severity of parkinsonian states was evaluated using a rating scale taking into account classical motor symptoms (bradykinesia, rigidity, tremor, freezing, posture, and arm posture), spontaneous activities (arm movements, spontaneous eye movements, and home cage activity), and other activities (vocalization, triggered eye movements, and feeding) (75).

### TH immunostaining and quantification of denervation

#### Brain sampling.

Rats were sacrificed by decapitation 1 day after the last session of sucrose self-administration and 1 hour after the last Pra or placebo injection (i.e., 6 weeks after 6-OHDA infusion or saline). Brains were immediately frozen in liquid nitrogen and stored at –80°C. They were then processed at –20°C for both immunostaining and HRMAS NMR to further match metabolomics and immunostaining-based lesions. For HRMAS NMR, thick sections of DS and Nacc tissue were pooled into disposable inserts and kept at –80°C until NMR analysis.

For immunostaining, 14 μm coronal sections of striatal levels of interest (19) were sampled and stored at –20°C. TH immunostaining and quantification of denervation were then carried out as previously described and as detailed in supporting information (20, 23). Human α-synuclein expression levels were revealed in the striatum by immunohistochemistry (see Supplemental Methods for protocol details).

### PD progression score in 6-OHDA model

In order to assign each 6-OHDA rat to a group mimicking a disease stage, as in clinics, we developed a score, referred to as the PDP score, that combined 3 criteria: (a) evaluation of neuropsychiatric symptoms (i.e., apathetic-like behavior), (b) evaluation of motor symptoms (i.e., fine motor deficit), both used in clinics, and (c) level of dopaminergic denervation in DS, only available postmortem in patients. Each criterion was ranked from 0 to 4 in integer values, as illustrated in Figure 2A. They were summed to produce the individual PDP score, ranging therefore from 0 to 12.

For the evaluation of behavioral deficits in the self-administration and stepping tests (criteria 1 and 2, respectively), we compared the performances of each animal before and after surgery. A value of 0 for each of these 2 criteria corresponded to an absence of deficit or to a deficit lower than 30%, i.e., the normal daily variability in the tests used. The following subcategories were set as follows: 1 = small deficit (30%–50%); 2 = medium symptoms (50%–70%); 3 = strong symptoms (70%–90%); and 4 = large or total deficit (>90%). Considering that motor symptoms appear after 70% of striatal dopaminergic neuron loss in PD patients, the value for dopaminergic denervation in the DS (criterion 3) was used as the limit between the prodromal-like and the clinical-like stages.

Regarding the PDP score obtained by addition of the values of the 3 criteria, 6-OHDA rats were classified as follows: (a) asymptomatic with weak lesions but no behavioral symptom (1 ≤ PDP score ≤ 2), (b) prodromal-like, i.e., presenting only neuropsychiatric disorders and limited DS lesions (3 ≤ PDP score ≤ 7), and (c), clinical-like, i.e., presenting neuropsychiatric disorders, motor symptoms, and widespread DS lesions (8 ≤ PDP score ≤ 12). All sham-operated animals scored 0, in spite of daily variability, as for PD-like animals (see above). Considering the low number of asymptomatic animals and the fact that they do not present any clinical relevance, they were excluded from the study.

### Human cohorts

Blood samples were obtained from de novo PD patients and matched-control subjects from the Parkinson’s Disease Biomarkers Program (PDBP) Consortium, supported by the NIND at the NIH and from Santa Lucia Foundation cohorts. Inclusion criteria for NIH patients included recent diagnosis (de novo diagnosed ≤ 1 year) of PD according to the criteria of the Movement Disorders Society (MDS) (2). For the Italian cohort, patients reporting symptoms for 3 years or less, according to MDS, were included (de novo patients ≤ 3 years). Thus, in all cohorts, patients had not received any antiparkinsonian treatment at the time of inclusion and sampling and presented no dementia or active psychiatric disorders nor any other medical conditions that could compromise the study. Inclusion criteria for controls were absence of neurological diseases, no family history of movement disorders, and no specific medical conditions.

The cohort from the NIH included 19 de novo PD patients without antiparkinsonian treatment, and 30 age- and sex-matched controls. The Santa Lucia cohort (first Italian cohort) included 21 de novo PD patients and 23 age- and sex-matched controls. A second set of 36 de novo PD patients from Santa Lucia was provided later to evaluate, in a prospective-like approach, the robustness of our method. All clinical and demographic information collected is summarized in Supplemental Table 1 (NIH and first Italian cohorts) and in Supplemental Table 3 (second Italian cohort). Moreover, 9 additional PD patients treated with Pra were also submitted to NMR analysis, but not used for statistical modeling.

### NMR experiments: 1H HRMAS NMR of 6-OHDA rat brain samples

#### Data acquisition.

All HRMAS NMR spectra were acquired, as previously described (76), using a Bruker Advance III spectrometer (IRMaGe, CEA) at 500 MHz. 1D spectra were acquired using a Carr-Purcell-Meiboom-Gill (CPMG) pulse sequence on spinning samples at 4 KHz and at T = 4°C.

#### Data processing.

Quantification was performed with jMRUI software based on a quantum estimation (QUEST) procedure (77). This procedure requires the use of a metabolite database and a complete assignment of spectra. Nineteen metabolites were assigned and quantified (see Figure 4A): acetate, alanine, ascorbate, choline, γ-aminobutyrate (GABA), glutamate, glutamine, glycine, glycerophosphocholine, glutathione, lactate, myo-inositol, *N*-acetylaspartate, PC, PCR, phosphoethanolamine, scyllo-inositol, and taurine. The amplitude of metabolite calculated by QUEST was normalized to the total spectrum signal. CRLB was calculated for each metabolite as estimates of the SD of the fit.

### 1H NMR of serum

#### Serum sampling.

For rats, blood was collected under gas anesthesia with isoflurane (2%) from the caudal vein after 2 hours of fasting and was stored in ice before being rapidly centrifuged at 1600*g* for 15 minutes at 4°C. The supernatant serum was removed and stored at –80°C until the day of NMR. The time before freezing never exceeded 30 minutes (78).

For monkeys, blood was collected under anaesthesia (0.05 mg/kg atropine i.m. followed by 15 mg/kg zoletil i.m.) from the saphenous vein after a 12-hour fast and submitted to the same protocol as rat blood. For humans, blood was collected preferably after 8 hours of fasting, or, if fasting was not feasible, after at least 8 hours on a low-fat diet, and stored at room temperature for 15 to 60 minutes before being centrifuged at between 1200*g* and 1500*g* at 4°C for 10 to 15 minutes. The supernatant serum was then removed and stored at –80°C. The day of NMR experiments, samples were slowly thawed on ice, then quickly centrifuged to eliminate possible cryoprecipitates. NMR tubes were filled with 60 μL serum sample and 120 μL PBS, 0.1 M in D_2_O (50% of D_2_O, pH = 7.4) and stored at 4°C until NMR acquisition.

#### Data acquisition.

All animal and human serum samples were submitted to the same NMR protocol. ^1^H NMR experiments were performed on a Bruker Advance III NMR spectrometer at 950 MHz (IBS) using a cryoprobe with a 3 mm tube holder. 1D spectra were systematically recorded using a CPMG pulse sequence.

#### Data processing.

The free induction decays were Fourier transformed and manually phased with the Bruker software Topspin, version 3.6.2. Then, further preprocessing steps (baseline correction, alignment, bucketing) were performed using NMRProcFlow, version 1.2, online (http://nmrprocflow.org).The spectra were segmented in 0.001 ppm buckets between 0 and 10 ppm, with exclusion of residual water peak, macromolecule signals, and other regions corresponding to pollution (5.38–5:30 ppm; 5.0–4.7 ppm; 2.2–2.0 ppm; 1.4–1.25 ppm; 1.15–1 ppm; 0.9–0.5 ppm). Each bucket was normalized to the total sum of buckets.

### Statistics

#### Multivariate analysis.

Data from liquid or HRMAS NMR were imported into SIMCA, version 14, for multivariate statistics. An unsupervised principal components analysis (PCA) was first used for global visualization of the distribution of all samples, followed by an OPLS to find discriminatory metabolites associated with a specific stage of the disease. For the latter, either a continuous (i.e., PDP score) or a discrete variable (i.e., disease-stage groups) was used to label each sample, leading in the case of the discrete variable to OPLS-DA (discriminant analysis). Scores were plotted in 2D versus the 2 first components of the OPLS models while loadings were plotted in 1D to mimic an NMR spectrum, but with positive and negative peaks indicating respectively up- and downregulated metabolites. Furthermore, in this “statistical spectrum,” each NMR variable was color coded according to its correlation with group belonging. Metabolites with a correlation of 0.5 or more were considered the most discriminant and were submitted to further univariate analysis.

The metabolites showing significant modification in at least 3 of the 4 PD models studied were submitted to multiple logistic regression using R software (version 3.6.1, R core team). Logistic regression was presented using a generic equation of the following form: log(*P*/[1 − *P*]) = *β_0_* + *β_1_* × *x*, where *x* represents metabolite relative amplitude and *β_0_* and *β_1_* are the parameters associated with the intercept and the degree of change in metabolites, respectively. For animals, the regressions, including crossvalidation, were conducted with prodromal-like animals (6-OHDA and α-synuclein prodromal-like rats) and corresponding sham-operated animals, while clinical-like animals were used for external validation (6-OHDA and α-synuclein clinical-like rats, and all primates). The same type of analyses was conducted using the NIH cohort (de novo patients and corresponding controls). The Italian data were used for external validation. For both animals and humans, all combinations of the 6 selected metabolites were tested, and subsequent ROC curves were generated for each of them to assess the quality of the fit using AUC and optimal threshold, which maximizes sensitivity and specificity. The accuracy, i.e., number of correctly predicted (true positive and true negative) divided by total sample, was calculated.

#### Univariate analysis.

All univariate analyses were performed using Graphpad Prism 8 software. All results were expressed as mean ± SEM, with a threshold for significance fixed at 0.05.

#### 6-OHDA models.

For self-administration and stepping tests, Pra effect univariate analysis was performed by applying repeated measure (RM) 1-way ANOVA followed by Šidák’s post hoc test. For histological and metabolomic data, 1-way ANOVA followed by Tukey’s post hoc test with correction for multiple comparisons was performed.

#### α-Synuclein models.

For the longitudinal α-synuclein study, some values were missing due to artifacts during NMR measurement. Data were therefore analyzed by fitting a mixed model proposed using a compound symmetry covariance matrix and were fitted using restricted maximum likelihood (REML). This model was followed by Tukey’s post hoc test.

#### MPTP monkeys.

Considering the low number of animals, the nonparametric Mann-Whitney test was used.

#### Humans.

Data were log transformed given the non-Gaussian distribution of metabolite levels. First, 1-tailed *t* tests were performed individually in the 2 different cohorts. Then we applied 2-way ANOVA with (a) origin of the cohort and (ii) group (control or PD) as factors followed by post hoc Šidák’s test.

### Study approval

Protocols complied with the European Union 2010 Animal Welfare Act, and the French directives were approved by the French national ethics committee (2013/113, no. 004) and by the local Grenoble and Lyon ethical committees (C2EA84 and CELYNE C2EA). For human samples, all donors gave written informed consent. For details concerning study approval in animals and humans, see Supplemental Methods.

## Author contributions

DM, TD, CC, SC, ELB, SB, and FF conducted research. DM, TD, CC, MD, and MBM conducted experiments. POF, VS, PB, and GS provided samples. DM, SB, and FF designed research. DM, SB, FF, and LV analyzed data. DM, SB, and FF wrote the manuscript.

## Supplementary Material

Supplemental data

ICMJE disclosure forms

## Version 1 12/16/2021

In-Press Preview

## Version 2 02/15/2022

Electronic publication
