# Supplementary material for: A metabolic biomarker predicts Parkinson’s disease at the early stages in patients and animal models
Source: J Clin Invest. 2022 Feb 15;132(4):e146400. doi: 10.1172/JCI146400 (PMC8843749; doi:10.1172/JCI146400)
Supplement: Supplemental data [file jci-132-146400-s142.pdf]

Supplemental information

Supplemental methods

6-OHDA bilateral injection

As previously described(20, 23), rats were subcutaneously injected with desipramine (15 mg/kg) 30 min before 6-OHDA injection in order to protect the noradrenergic neurons. All animals were then anesthetized by intraperitoneal injection of Ketamine-Xylazine (100-7 mg/kg), and placed in a stereotactic frame (Kopf instrument).

Animals received a bilateral injection of 2.3 µl 6-OHDA (3 mg/ml) or NaCl 0.9% (sham), at a flow rate of 0.5 µl/min according to the stereotaxic atlas of Paxinos and Watson(80) and relative to bregma: incisor bar placed at -3.2 mm: anteroposterior (AP) = -5.4 mm / lateral (L) = +/- 1.8 mm / dorsoventral (V) = -8.1 mm. For lesioned animals presenting transient starvation 2–3 days after surgery, supplementation with a high-caloric liquid diet and palatable food was implemented for 1–2 weeks, and stopped 10 days before serum sampling. Animals that did not recover were excluded.

Alpha synuclein bilateral injection

As previously described (21), rats were anesthetized with isoflurane 2.5% and placed in a stereotaxic frame (Kopf Instruments) in order to receive bilateral injections of AAV-hA53Tα-syn (1µl-7.0×1012 vg/ml) or AAV-GFP (7.0 ×1012 vg/ml, sham animals) in the SNc (AP = -5.1 mm and -5.6 mm / L = +/- 2.2 mm / V = 8 mm from bregma).

Operant self-administration (motivational component)

This task was carried out with a fixed ratio of 1, a single support on the reinforced lever resulting in the deliverance of one reward. Each session ended when 100 rewards were obtained, or at the end of the allotted time (1 hour). The number of rewards obtained was counted for each session by MED-PC IV software.

Stepping test (motor component)

The test was carried out three times by two different experimenters, blind to the experimental conditions.

TH-immunostaining and quantification of striatal DA denervation

After post-fixation with paraformaldehyde 4%, striatal slices were incubated with an anti-TH antibody (mouse monoclonal MAB5280, Millipore, France, 1: 2500) overnight at 4°C. Then slices were incubated with biotinylated goat anti-mouse IgG antibody (BA-9200, Vector Laboratories, Burlingame, CA, USA; 1: 500). Avidin-peroxidase conjugate revealed immunoreactivity (Vectastain ABC Elite, Vector Laboratories Burlingame, CA, USA).

Quantification of the extent of striatal dopaminergic denervation was determined with ICS FrameWork computerized image analysis system (Calopix, 2.9.2 version, TRIBVN, Châtillon, France) coupled with a light microscope (Nikon, Eclipse 80i). After drawing masks from three striatal levels, optical densities (OD) were measured for each striatal sub-region (DS and Nacc). OD were expressed as percentages relative to the mean optical density obtained from the homologous regions of sham-operated animals.

TH-immunostaining human α-synuclein expression

Human α-synuclein expression levels were revealed in the striatum by immunohistochemistry. The selected sections of the striatum were incubated with a mouse monoclonal antibody raised against human α-synuclein (clone syn211 Thermo Scientific, MA5-12272, 1:1000) overnight at room temperature and revealed by an anti-mouse peroxidase EnVisionTM system (DAKO, K4007) followed by DAB incubation.

^{1}H HRMAS NMR in 6-OHDA rat brain samples

Just before HRMAS analysis, 10 µl D_2O was added to the inserts containing the brain biopsies, which was then sealed and packed into a 4 mm zirconia MAS rotor.

One-dimension spectra were acquired using a Carr-Purcell-Meiboom-Gill (CPMG) pulse sequence with a total echo time (TE) of 30 ms and 250 µs interpulse delay. The residual water signal was pre-saturated during 1.7 s of relaxation. Each spectrum using 256 averages lasted 17 minutes).

^{1}H NMR of serum

One-dimension spectra were systematically recorded using a CPMG pulse sequence for edition of metabolite, with TE = 120 ms and 250 µs interpulse delay. The residual water signal was pre-saturated during 2 seconds of relaxation. Each spectrum using 128 averages lasted 10 minutes.

Assignment of peaks was performed using 2-dimension homonuclear ^{1}H-^{1}H (TOCSY) and heteronuclear ^{1}H-^{13}C (HSQC) spectra of selected samples, and database. When necessary, addition of selected metabolites was used to unravel ambiguous assignments.

Multivariate statistics

For OPLS/OPLS-DA, the total number of components was determined using the cross-validation procedure, which produces the R2Y and Q2 factors that indicate respectively the goodness of the fit and the predictability of the model. A model is considered as robust and predictive when both factors were ≥ 0.5.

Furthermore, analysis of variance of cross-validated predictive residuals (CV-ANOVA) was used to assess the significance of the model.

Study approval

Protocols complied with the European Union 2010 Animal Welfare Act and the French directive 2010/63, and were approved by the French national ethics committee (2013/113) n° 004 and by local ethical committee C2EA84 and CELYNE C2EA.

Each PDBP study site's local IRB approved study protocols, and all participants provided written informed consent for participation in PDBP. As one goal of the PDBP is to provide a biorepository of samples from a well-characterized set of individuals, participants consented to sharing of samples and deidentified data with investigators approved by the Biospecimen Review Access Committee at the time of enrollment.

Supplemental data

Supplemental tables

Supplemental Table 1

Supplemental Table 1: Clinical characteristics of PD patients

Patient	Cohort	Sex	Age (Years)	Duration (months)	H&Y	UPDRS.I	UPDRS.II	UPDRS.III	Treatment
1	NIH	F	59	10	0	8	6	4	N/A
2	NIH	M	65	8	2	15	11	20	N/A
3	NIH	M	63	1	1	2	11	14	N/A
4	NIH	M	63	2	3	13	10	36	N/A
5	NIH	M	68	8	2	-	-	38	N/A
6	NIH	M	71	7	2	6	6	29	N/A
7	NIH	F	62	5	2	7	4	38	N/A
8	NIH	F	51	3	2	6	11	16	N/A
9	NIH	M	58	10	2	5	15	13	N/A
10	NIH	M	69	7	2	-	-	17	N/A
11	NIH	F	51	1	4	-	-	45	N/A
12	NIH	F	53	9	2	4	2	14	N/A
13	NIH	F	58	1	2	14	6	10	N/A
14	NIH	F	52	8	2	5	8	10	N/A
15	NIH	M	62	0	1	3	7	12	N/A
16	NIH	M	43	7	1	4	5	12	N/A
17	NIH	F	75	2	2	9	8	12	N/A
18	NIH	M	78	7	1	16	8	6	N/A
19	NIH	F	53	5	2	8	8	17	N/A
20	Italy first	F	68	12	2	-	-	10	N/A
21	Italy first	M	53	12	1	-	-	10	N/A
22	Italy first	M	71	24	1.5	-	-	10	N/A
23	Italy first	-	-	-	-	-	-	-	N/A
24	Italy first	F	67	12	1.5	-	-	8	N/A
25	Italy first	M	44	12	1	-	-	8	N/A
26	Italy first	F	64	12	2	-	-	10	N/A
27	Italy first	-	-	-	-	-	-	-	N/A
28	Italy first	M	68	-	-	-	-	-	N/A
29	Italy first	F	54	24	-	-	-	6	N/A
30	Italy first	F	65	12	1	-	-	6	N/A
31	Italy first	F	71	24	-	-	-	14	N/A
32	Italy first	M	63	24	-	-	-	24	N/A
33	Italy first	M	59	12	1.5	-	-	4	N/A
34	Italy first	M	61	-	-	-	-	14	N/A
35	Italy first	M	66	0	1.5	-	-	14	N/A
36	Italy first	M	76	12	1	-	-	1	N/A
37	Italy first	M	72	12	-	-	-	24	N/A
38	Italy first	M	42	24	-	-	-	26	N/A
39	Italy first	M	58	0	1.5	-	-	10	N/A
40	Italy first	F	49	36	-	-	-	9	N/A
41	Italy first	M	67	12	1	-	-	3	N/A
42	Italy first	M	57	-	1	-	-	2	N/A
A	NIH	M	66	0	2	-	-	31	Pra
B	NIH	M	69	10	2	-	-	43	Pra
C	NIH	F	62	1	2	-	-	45	Pra
D	NIH	M	82	11	2	17	18	22	Pra
E	NIH	M	62	4	2	17	13	11	Pra
F	NIH	M	63	7	2	5	1	15	Pra
G	NIH	M	69	3	2	7	12	21	Pra
H	NIH	F	58	10	2	13	3	9	Pra
I	NIH	M	70	5	3	10	7	30	Pra

F = Female; M = Male; Duration = Duration from diagnosis (NIH) or symptoms onset (Iitaly) ; H&Y = Hoehn and Yahr scale; UPDRS = MDS - Unified Parkinson disease rating scale; Pra = Pramipexole; N/A = not applicable

Table S1: Clinical characteristics of PD patients

Supplemental Table 2

Metabolite	Group	^{1}H ppm	^{13}C ppm	Multiplicity
3-hydroxybutyrate	ɣ-CH_3	1.19	24,4	d
	half α-CH_2	2.30	49,2	dd
	half α-CH_2	2.39	49,2	dd
	β-CH	4.15		m
Acetoacetate		3,44		s
		2.27		s
Acetate	CH_3	1.91	25.9	s
Acetone	CH_2CO	2.22	32,9	s
Alanine	CH_3	1.47	18.8	d
	α-CH	3.77	53.5	q
Albumin lysyl	ϵ-CH_2	2.88	42.0	t
	ϵ-CH_2	2.95	41.9	t
	ϵ-CH_2	3.01	42.0	t
Arginine	ɣ-CH_2	1.64		
	ɣ-CH_2	1.69		
	β-CH_2	1.89		
	δ-CH_2	3.23	43.2	t
Aspartate	half β-CH_2	2.66		q
	half β-CH_2	2.80		dd
Betaine	CH2	3,9		s
	CH_3	3.26		s
Cholesterol	C18 (in HDL)	0.66		m
	C18 (in VLDL)	0.68		
	C26 and C27	0.83	25.2	m
Choline	N $(CH_3)_3$	3.21	56.7	s
		3.51		
		4.06		
Citrate	half CH_2	2.53		d
	half CH_2	2.66		d
Creatine	CH_3	3.03		s
	CH_2	3.92		s
Creatinine	CH_3	3.04		s
	CH_2	4.05		s
Dimethylamine	CH_3	2.71		s
Ethanol	CH_3	1.17	21.6	t
	CH_3COH	3.65		q
Fatty acids (mainly LDL)	$CH_3(CH_2)n$	0.84	16.5	m
	$(CH_2)n$	1.27	32.2	m
Fatty acids (mainly VLDL)	$CH_3CH_2CH_2C=$	0.86		m
	CH2CH2CO	1.57	27.4	m
	$CH_2CH_2CH_2CO$	1.29		m

Fatty acids	CH_3CH_2	0.93	21.05	m
	$CH_3CH_2(CH_2)n$	1.24	34.4	m
	$CH_3CH_2(CH_2)n$	1.26	25.2	m
	CH_2	1.26	19.2	m
	CH_2	1.30		m
	$CH_2CH_2C{=}C$	1.68	29.2	

Metabolite	Group	^{1}H ppm	^{13}C ppm	Multiplicity
Fatty acids	$CH_2C{=}C$	2.00	29.7	m
	CH_2CO	2.22	36.3	m
	$C{=}CCH_2C{=}C$	2.72	28.1	m
	CH=CHCH2CH=CH	5.26	130.6	m
	CH=CHCH2CH=CH	5.29	132.2	m
Formate	CH	8.45		s
Fructose		3,99		m
		4.01		dd
Fucose / β-Galactose		4.54		d
Glucose	H2	3.24	76.9	t
	H4	3.40	72.4	t
	H4	3.41	72.4	t
	H5	3.46	78.6	m
	H3	3.48	78.5	t
	H2	3.53	74.3	q
	H3	3.71	75.6	t
	half CH_2-C6	3.72	63.5	q
	half CH_2-C6	3.76	63.4	m
	H5	3.82	74.2	ddd
	half CH_2-C6	3.84	63.4	m
	half CH_2-C6	3.89	63.5	dd
	H1	4.64	98.7	d
	H1	5.23	94.9	d
Glutamate	half β-CH_2	2.04		m
	half β-CH_2	2.12		m
	half γ-CH_2	2.34	33.9	m
	half γ-CH_2	2.36		m
		3.74		m
Glutamine		2.08		
		2.09		
	half β-CH_2	2.11	29.7	m
	half γ-CH_2	2.44	33.9	m
		2.46	57.4	m
		3.74		
Glycerol	half CH_2	3.56	65.8	q
	half CH_2	3.65	65.6	q
	C_2-H	3.87	74.6	m
Glycerophosphocholine		3.22		s
	NCH_2	3.66	68.7	m

	OCH_2	4.29	62.2	m
Glycerol backbone		4.06		

Metabolite	Group	^{1}H ppm	^{13}C ppm	Multiplicity
PGLYs and TAGs	CHOCOR	4.22		
		5.20		
Glycine	CH_2	3.55	44.3	s
Histidine		3.09		dd
		3.98		dd
	H4	7.04		s
	H2	7.75		s
Isoleucine	δ-CH_3	0.93	13,9	t
	β-CH3	1.00	17,15	d
	half γ-CH_2	1.24		
	half γ-CH_2	1.46		
		1.96		
		3.65		
Lactate	CH_3	1.32	22.7	d
	CH	4.11	71.2	q
Lactose		3.55		
		3.66		
		3.97		
		4.45		
Leucine	δ-CH_3	0.95		d
	δ-CH_3	0.96		d
		1.66		m
		1.70	42.7	m
		1.73		m
	α-CH	3.71		
Lysine	γ-CH_2	1.43		m
	γ-CH_2	1.49		m
	δ-CH_2	1.72		m
	β-CH_2	1.88		m
	β-CH_2	1.91		m
		3.02		t
		3.74		t
Mannose		4.89		d
		5.18		d
Methanol	CH_3OH	3.35		s
Methionine		2,64	31,4	t
Methionine		2.15		s
Myo-inositol		4.05		
		3.27		t
N-acetyl-glycoprotein 1	$NHCOCH_3$	2.04	24.7	s
N-acetyl-glycoprotein 2	$NHCOCH_3$	2.07	25	s

Metabolite	Group	^{1}H ppm	^{13}C ppm	Multiplicity
Phenylalanine	half β-CH_2	3.26		
	α-CH	3.97		
	H2, H6	7.31		d
	H4	7.35		m
	H3, H5	7.40		m
Proline	ɣ-CH_2	1.98		m
	ɣ-CH_2	2.01		m
	half β-CH_2	2.05		m
	half β-CH_2	2.34		m
	half δ-CH_2	3.33		m
	α-CH	4.12		m
Succinate		2.39		s
Threonine	ɣ-CH_3	1.31		d
	α-CH	3.55		d
	β-CH	4.23		m
Trehalose		3.40	74.3	
Tyrosine		6.88		d
	H2, H6	7.18		d
Valine	CH3	0.98	19.4	d
	CH3	1.03	20.6	d
	β-CH	2.26		m
	α-CH	3.60	63.4	d
Urea	$NH_2C{=}ONH_2$	5.77		
Xylose		3.41	78.6	

Table S2: List of metabolites identified in serum samples.
For each metabolite, chemical group, assignment for ^{1}H and ^{13}C and multiplicity of peak are presented.
Multiplicity: singulet (s), doublet (d), doublet doublet (dd), multiplet (m)

Supplemental Table 3

Supplemental Table 3: Clinical characteristics of additional PD patients

Patient	Cohort	Sex	Age (Years)	Duration (months)	H&Y	UPDRS.I	UPDRS.II	UPDRS.III	Treatment
1	Italy second	M	72	<12	1,5	-	-	9	N/A
2	Italy second	M	69	12-18	1	-	-	6	N/A
3	Italy second	F	48	12-18	1	-	-	5	N/A
4	Italy second	M	55	<12	1	-	-	9	N/A
5	Italy second	M	83	12-18	1,5	-	-	12	N/A
6	Italy second	M	80	<12	1	-	-	17	N/A
7	Italy second	F	55	<12	1,5	-	-	23	N/A
8	Italy second	M	60	<12	1,5	-	-	12	N/A
9	Italy second	F	65	<12	1	-	-	3	N/A
10	Italy second	M	44	<12	1	-	-	7	N/A
11	Italy second	M	55	12-18	2	-	-	18	N/A
12	Italy second	M	73	<12	1	-	-	23	N/A
13	Italy second	F	75	12-18	1	-	-	12	N/A
14	Italy second	F	64	12-18	1	-	-	7	N/A
15	Italy second	M	75	12-18	2	-	-	13	N/A
16	Italy second	M	46	12-18	1	-	-	1	N/A
17	Italy second	M	63	12-18	1,5	-	-	25	N/A
18	Italy second	M	63	12-18	1	-	-	15	N/A
19	Italy second	M	58	12-18	2,5	-	-	19	N/A
20	Italy second	M	50	12-18	1	-	-	10	N/A
21	Italy second	F	61	12-18	1,5	-	-	7	N/A
22	Italy second	M	38	<12	1,5	-	-	10	N/A
23	Italy second	F	76	12-18	1,5	-	-	21	N/A
24	Italy second	M	60	12-18	1	-	-	4	N/A
25	Italy second	F	56	<12	1	-	-	8	N/A
26	Italy second	F	56	12-18	1	-	-	7	N/A
27	Italy second	F	73	<12	1	-	-	7	N/A
28	Italy second	M	69	<12	1,5	-	-	18	N/A
29	Italy second	M	50	<12	1	-	-	22	N/A
30	Italy second	M	48	12-18	1	-	-	11	N/A
31	Italy second	F	53	<12	1	-	-	10	N/A
32	Italy second	M	79	12-18	1	-	-	18	N/A
33	Italy second	F	53	12-18	1	-	-	11	N/A
34	Italy second	M	63	<12	1	-	-	10	N/A
35	Italy second	M	77	12-18	2	-	-	20	N/A
36	Italy second	M	71	12-18	2,5	-	-	22	N/A

F = Female; M = Male; Duration = Duration from symptoms onset ; H&Y = Hoehn and Yahr scale;
UPDRS = MDS - Unified Parkinson disease rating scale; Pra = Pramipexole; N/A = not applicable

Table S3: Clinical characteristics of additional PD patients from Italy (Second cohort)

Supplemental figures

Supplemental Figure 1

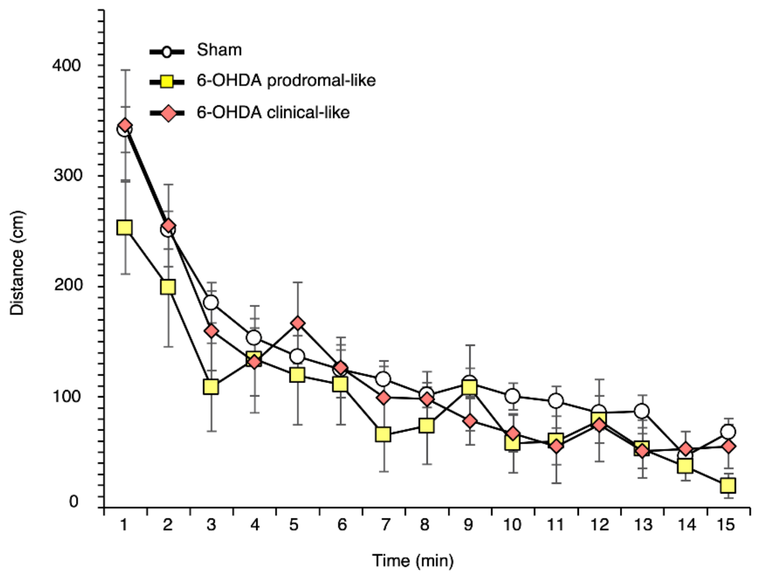

Figure S1: Ambulatory motor activity measured in an open field.
Dopaminergic lesions did not affect horizontal ambulatory activity measuring in an open area for 15-min.
Data are presented by mean value ± SEM for each minute.

Supplemental Figure 2

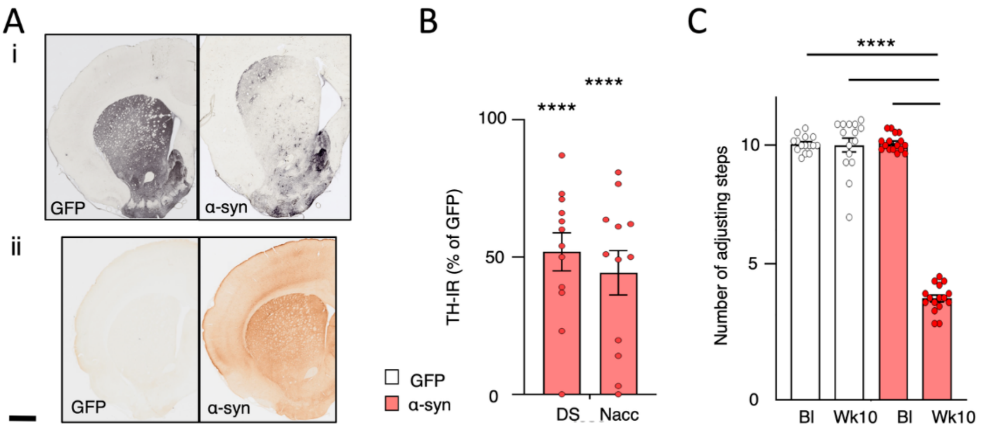

Figure S2: AAV-hA53Tα-syn vector-mediated overexpression of alpha-synuclein in rat SNc, induces striatal dopaminergic degeneration associated with α-syn overexpression and leads to fine motor dysfunction.

(A-B) Examples of coronal sections of GFP and α-syn rat brains stained for TH (A) and human α-syn (B). Scale bar represents 1mm.
(C) Quantification of TH-IR staining at the striatal level, expressed as percentage of the mean value obtained for GFP animals (n = 12). We observe a large decrease of TH-positive neurons in DS after 10 weeks in alpha-synuclein animals (n = 12).
(D) α-syn injection reduced the number of adjusting steps in a stepping procedure after 9 weeks. Results are expressed as the mean number of forelimb adjustments for two trials at baseline (left bar) and 9 weeks (right bar) for GFP (n=12) and alpha-synuclein animals (n=12).
BL: Baseline ; Wk10: Week 10 ; α-syn: alpha synuclein

Means ± SEM ****: $p \leq 0.0001$, Two-way ANOVA or RM-ANOVA followed by post-hoc Tukey test.

Supplemental Figure 3

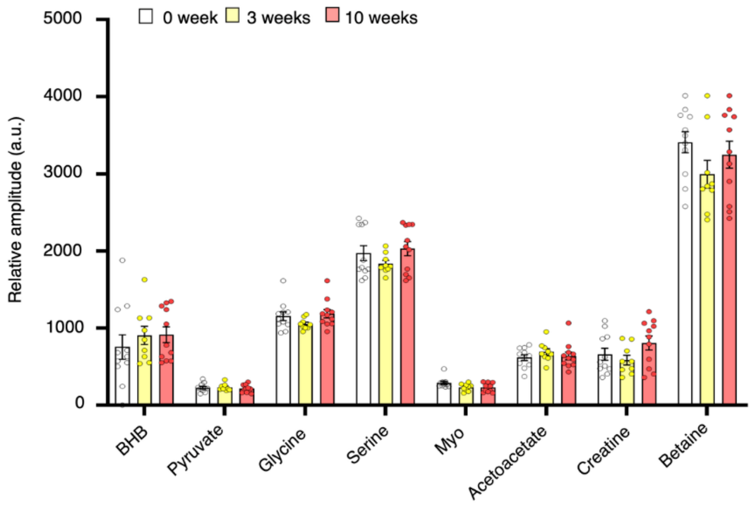

Figure S3: GFP viral infusion induces metabolic dysregulation in serum samples. Representative histogram showing relative quantitative variations of signal between samples from GFP animals for acetoacetate, betaine, BHB, creatine, glycine, myo-inositol, pyruvate, serine, the key metabolites implicated in discrimination of 3 groups in OPLS-DA of alpha synuclein animals. White bar corresponds to samples at week 0 (n = 11), yellow to 3 weeks post GFP infusion (n = 9) and red to after 10 weeks (n = 11). Data are presented as mean values ± SEM and tested by one-way ANOVA followed by posthoc test of Tukey.

Supplemental Figure 4

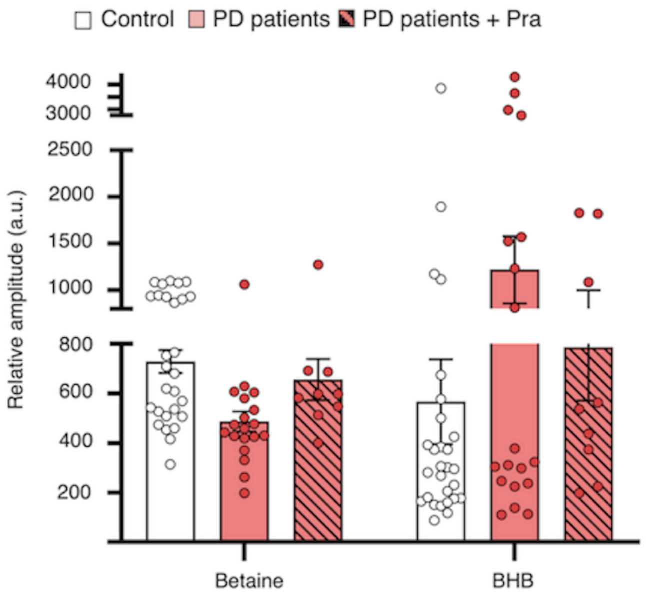

Figure S4: BHB and betaine levels are partially normalized in NIH PD patients treated with pramipexole.

Representative histogram showing relative quantitative variations of signal between Control (white bar, n = 29), PD patients (red bar, n = 19) and PD patients treated with Pra (hatched bar, n = 9) for BHB and betaine. Data are presented as mean values ± SEM.

Supplemental Figure 5

A

$$\text{logit}(P) = \log(P/(1 - P))$$

$$= 132.29 - 0.45\ BHB - 0.82\ Pyruvate + 0.36\ Valine + 0.17\ Acetoacetate - 0.39\ Creatine + 0.07\ Betaine$$

B

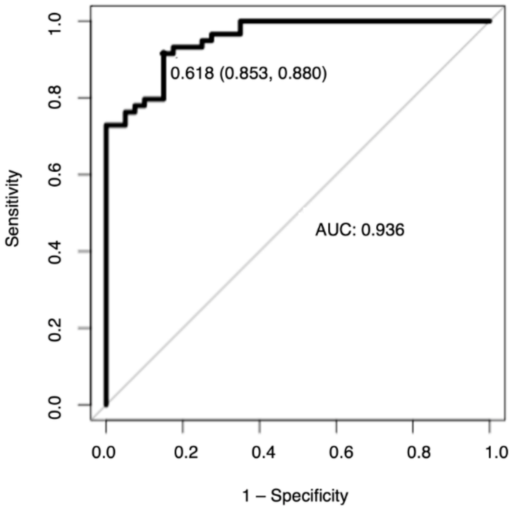

Figure S5: Logistic regression curve for panel of serum metabolites included: BHB, acetoacetate, valine, creatine, betaine and pyruvate.

(A) ROC curve from serum samples of all PD-like animal models. AUC = 0.954, sensitivity: 0.85, specificity: 0.915.

(B) Algorithm of logistic regression for all PD-like animals (n = 48) vs shams (n = 39).
